# Gold–graphene quantum dot hybrid nanostructures for solar-to-chemical energy conversion: interfacial charge dynamics, hydrogen evolution, and water splitting

**DOI:** 10.1039/d6ra06585f

**Published:** 2026-10-26

**Authors:** Chou-Yi Hsu, Rosull Saadoon Abbood, Hüseyin Kurt, Wade Ghribi, I. B. Sapaev, Man Mohan Shukla, Harvinder Singh Sohal, Aseel Smerat, Taraneh Hieunaz Chavoushi

**Affiliations:** a Department of Innovation, Yuan An BioResearch & Technology Co., Ltd Tainan 718004 Taiwan; b Medical Laboratory Techniques Department, College of Health and Medical Technology, University of Al-maarif Anbar Iraq; c Department of Electrical and Electronics Engineering, Faculty of Engineering, Istanbul Aydin University Istanbul Türkiye; d Department of Computer Engineering, College of Computer Science, King Khalid University Al-Faraa Kingdom of Saudi Arabia; e Head of the Department Physics and Chemistry, Tashkent Institute of Irrigation and Agricultural Mechanization Engineers National Research University Tashkent Uzbekistan; f Scientific Researcher of the University of Tashkent for Applied Science Uzbekistan; g School of Engineering, Central Asian University Tashkent 111221 Uzbekistan; h Western Caspian University, Scientific Researcher Baku Azerbaijan; i Department of Computer Science and Engineering, Pranveer Singh Institute of Technology Kanpur UP India; j Department of Chemistry, University Institute of Sciences, Chandigarh University Mohali Punjab India; k Hourani Center for Applied Scientific Research, Al-Ahliyya Amman University Amman 19328 Jordan; l Department of Biosciences, Saveetha School of Engineering, Saveetha Institute of Medical and Technical Sciences Chennai 602105 India; m Young Researchers and Elite Club, Tehran Branch, Islamic Azad University Tehran Iran thanhhieunchauu@gmail.com

## Abstract

Gold–graphene quantum dot (Au–GQD) hybrid nanostructures provide a versatile platform for coupling plasmonic light harvesting, quantum-confined carbon states, and interfacial redox activity in solar-to-chemical energy conversion. However, the field remains mechanistically fragmented, and the roles of Au, GQDs, and their evolving interface are often insufficiently distinguished. This review establishes a unified framework linking electronic structure, surface chemistry, interfacial coupling, photon utilization, excited-state partitioning, carrier transport, and surface redox turnover. It critically analyzes how Au size, GQD surface states, heteroatom functionality, contact geometry, and energetic alignment govern structure–property–performance relationships. Available evidence for hydrogen evolution and water splitting is examined alongside supporting studies on plasmonic absorption, hot-electron transfer, and type-II charge separation, while distinguishing direct solar-fuel conversion from indirect optical or photoelectronic responses. The synthesis reveals that productive performance depends not on simple component coexistence, but on function-specific interfacial organization and preservation of redox-competent carriers. Major limitations include inconsistent material identity, structural reconstruction, nonstandardized performance metrics, limited *operando* validation, and uncertain scalability. The review concludes with design priorities for stable, low-Au, spatially organized architectures and standardized evaluation pathways toward reproducible solar-fuel systems. This is the first dedicated review focusing specifically on Au–GQD hybrid nanostructures for solar-to-chemical energy conversion, providing an integrated analysis of their interfacial physicochemical properties and functional roles.

## Abstract 2

Au–GQD interfaces couple light harvesting with charge transfer and redox chemistry. This review links interfacial design to hydrogen generation and water splitting, while identifying gaps in mechanistic evidence and operational stability.

## Graphical abstract

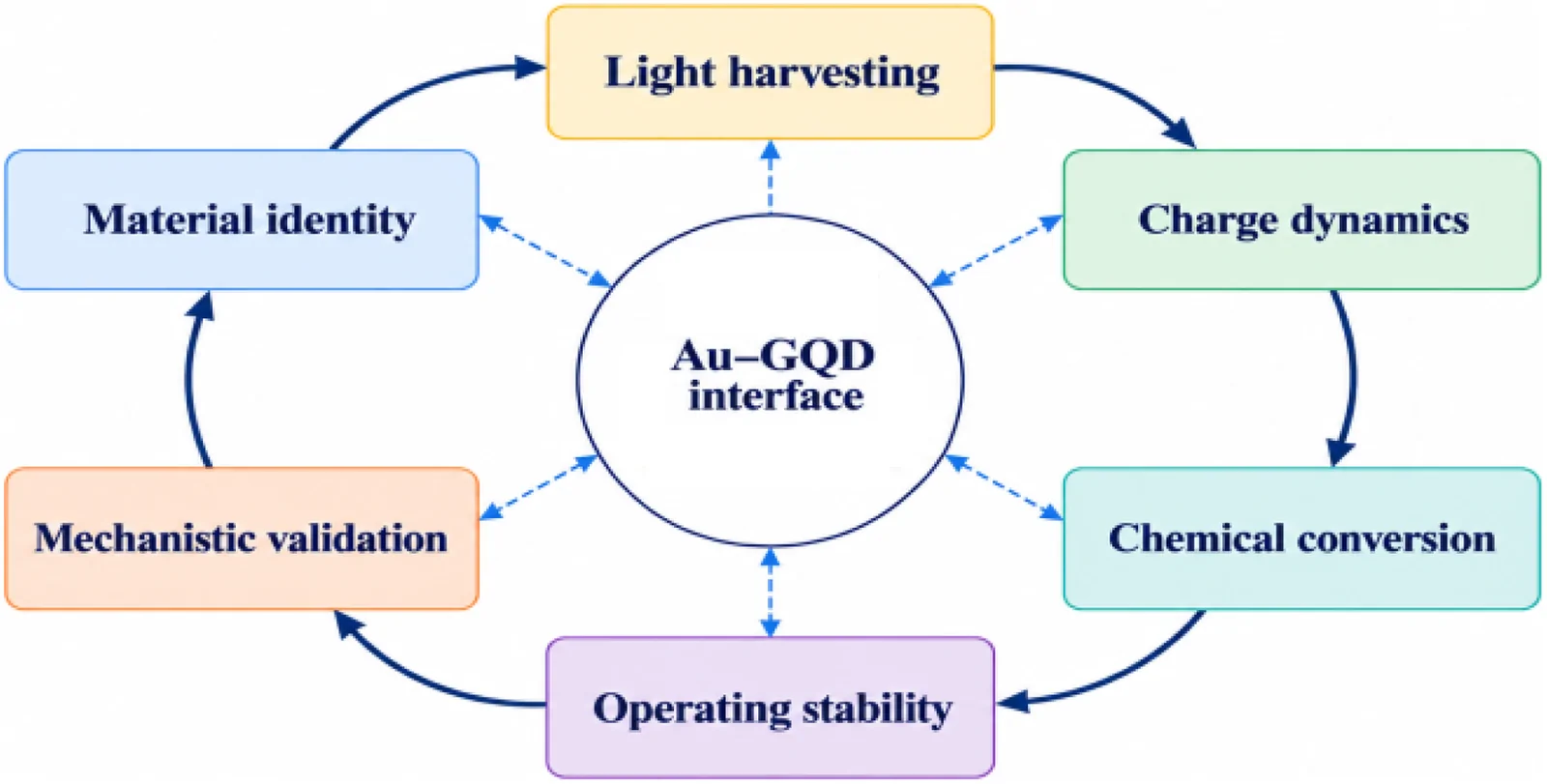

## 1. Introduction

The conversion of solar energy into storable chemical fuels remains a central challenge in the transition toward low-carbon energy systems. Unlike photovoltaic electricity generation, solar-to-chemical conversion must integrate photon absorption, excited-state formation, charge separation, carrier transport, interfacial redox chemistry, and product release within a single functional architecture.^1–4^ Losses at any stage can dominate the overall process, making material composition alone an insufficient predictor of performance. The design of effective solar-fuel systems therefore requires control over the spatial and energetic coupling between light-harvesting domains, charge-transport pathways, and catalytic sites.^5,6^ Hybrid nanostructures are particularly relevant in this context because they can combine functions that are difficult to realize within a single material, provided that their interfaces are electronically selective and chemically stable.^7,8^

Gold–graphene quantum dot (Au–GQD) hybrid nanostructures represent a distinctive platform for such integration. Nanoscale gold can act as a plasmonic absorber, hot-carrier source, electron donor, catalytic site, or structural template, depending on its size, morphology, coordination environment, and surrounding dielectric medium.^9–11^ GQDs contribute size-dependent electronic states, chemically addressable edges, tunable surface functionality, and pathways for charge acceptance or transport. Their combination can therefore create interfaces capable of linking optical excitation with chemical turnover. However, this functional complementarity is not intrinsic to the simple coexistence of Au and GQDs. Productive coupling depends on energy-level alignment, interfacial bond identity, contact geometry, surface-state distribution, carrier lifetime, and accessibility of the redox-active surface.^12–15^ The same interface may promote charge separation under one structural configuration but introduce trapping, quenching, or recombination under another.

The scientific importance of this field extends beyond the search for higher hydrogen-generation rates. Au–GQD systems provide a model platform for examining how plasmonic excitation, quantum-confined carbon states, and heterogeneous surface chemistry interact across nanoscale interfaces. They also expose unresolved questions that are broadly relevant to solar-fuel materials. It remains difficult to distinguish near-field enhancement from hot-electron injection, energy transfer from net charge transfer, beneficial trapping from terminal carrier localization, and stable hybridization from light-induced structural reconstruction.^16–19^ In addition, the term “Au–GQD hybrid” is applied to structurally different systems, including nanoparticle–dot assemblies, nanocluster–dot interfaces, grafted photoelectrodes, core–shell-like architectures, and carbon structures derived from transformed GQD precursors.^20,21^ Without resolving these distinctions, apparently similar materials may be compared despite operating through different mechanisms.

Previous review articles have provided valuable summaries of GQDs in energy-related applications; however, their scopes and analytical approaches differ from the present review. Reviews focusing on GQD-based heterogeneous catalysts mainly summarized synthesis strategies, surface modification approaches, and applications in overall water splitting and metal–air batteries, emphasizing the catalytic potential of GQDs derived from their surface area, active sites, and electron transport capability.^22^ However, these studies primarily considered GQDs as catalytic components and did not extensively examine how metal–GQD interfacial structures regulate electronic coupling, excited-state evolution, and charge utilization. Similarly, broader reviews on graphene-based materials for hydrogen production evaluated various graphene derivatives, including graphene oxide, reduced graphene oxide, metal oxide/graphene composites, and GQDs, but their discussion remained focused on material categories rather than the mechanistic determinants controlling a specific metal–quantum dot interface.^23^ Reviews covering carbon and GQDs in energy and optoelectronic devices further highlighted quantum confinement, optical properties, and functionalization strategies, while also identifying challenges related to stability, scalability, and cost; however, the connection between interfacial physicochemical characteristics and chemical conversion pathways was not deeply developed.^24^ In addition, recent reviews on GQD-assisted photocatalytic CO_2_ reduction and energy storage applications have provided detailed discussions of surface engineering, charge separation, and device performance, but mainly within individual application domains rather than through an integrated structure–interface–reaction framework.^25,26^

In contrast to previous reviews, the present work focuses specifically on Au–GQD hybrid nanostructures and develops a mechanistic understanding of how interfacial properties govern solar-to-chemical conversion. Rather than considering Au incorporation only as a strategy for improving optical absorption or catalytic activity, this review evaluates the coupled influence of Au size, GQD surface states, heteroatom functionalization, bond configuration, contact geometry, and electronic alignment on charge generation, transport, and redox processes. Furthermore, this review distinguishes between direct evidence of solar-fuel formation and indirect optical or photoelectronic observations, addressing an important limitation in the interpretation of Au–GQD systems. By integrating interface chemistry, electronic structure, excited-state dynamics, and reaction-level performance, this work provides a framework for understanding the conditions under which Au–GQD coupling contributes to effective solar-to-chemical energy conversion and when the observed enhancement mainly originates from optical or electronic effects rather than productive chemical transformation.

## 2. Physicochemical basis of Au–GQD hybridization: electronic structure, surface chemistry, and interfacial coupling

### 2.1. Electronic structure across the metal–quantum-dot boundary

The physicochemical identity of an Au–GQD hybrid originates from the interaction between two electronically dissimilar components. Gold nanoparticles exhibit a quasi-continuous density of electronic states, a high concentration of mobile carriers, and a Fermi level governed by particle size, surface coordination, crystallographic orientation, and the surrounding dielectric environment. GQDs, in contrast, possess discrete or broadened electronic levels arising from quantum confinement, finite lateral dimensions, edge termination, structural disorder, and localized surface states. Their electronic structure cannot therefore be represented solely by the idealized valence- and conduction-band picture used for bulk semiconductors. Instead, the accessible states comprise contributions from confined π-domains, edge-localized orbitals, heteroatom-induced levels, oxygen-containing groups, and defect-related traps.

Hybridization alters these electronic structures through electrostatic equilibration and orbital interaction. Before contact, Au and GQDs may possess different work functions and chemical potentials. Once coupled, charge redistribution proceeds until an interfacial equilibrium is established, producing band bending, local dipoles, and shifts in the effective Fermi level.^27–29^ The direction and magnitude of this redistribution are not fixed material constants because the work function of GQDs is strongly dependent on surface chemistry and structural heterogeneity. Likewise, the electronic behavior of nanoscale Au can deviate from that of bulk gold when the particle dimensions approach the mean free path of electrons or when a substantial fraction of atoms occupy low-coordination surface sites.

The resulting interface should therefore be interpreted as an electronically reconstructed region rather than a simple physical contact between a metal and a carbon nanodot. The degree of reconstruction determines whether the two components retain largely independent electronic identities or form strongly coupled states with partial orbital hybridization. This distinction is fundamental because weakly coupled assemblies support localized component-specific states, whereas stronger coupling can broaden GQD levels, modify Au surface states, and create new interfacial pathways.^30–32^ Accordingly, the first conceptual requirement is to treat the Au–GQD boundary as an emergent electronic phase whose properties depend on both components and on the manner in which they are joined. This electronic basis leads directly to the role of surface chemistry in defining the actual nature of that junction.

As illustrated in Fig. 1, the formation of the GQD–Au hybrid begins with the light-assisted carbonization and nucleation of glucose-derived species, followed by surface growth into oxygen-functionalized GQDs containing hydroxyl, carbonyl, carboxyl, and C–O-related groups. These surface functionalities provide chemically active sites for interaction with the added HAuCl_4_ precursor and promote the nucleation and attachment of Au domains onto the GQD surface, producing a self-passivated metal–quantum-dot interface.^31^ From the perspective of electronic structure, this intimate contact brings the discrete, surface-sensitive electronic states of the GQDs into proximity with the quasi-continuous states of metallic Au, thereby creating the structural basis for charge redistribution, local dipole formation, and possible modification of the effective Fermi level after equilibration. The schematic therefore emphasizes that the resulting Au–GQD boundary is not merely a physical assembly, but an interfacial region whose electronic behavior is determined by the GQD surface chemistry, Au nucleation geometry, contact distance, and degree of electronic coupling. Nevertheless, the scheme represents a proposed formation pathway and does not by itself directly prove Fermi-level equilibration or orbital hybridization, which would require complementary spectroscopic or electronic measurements.

**Fig. 1:**
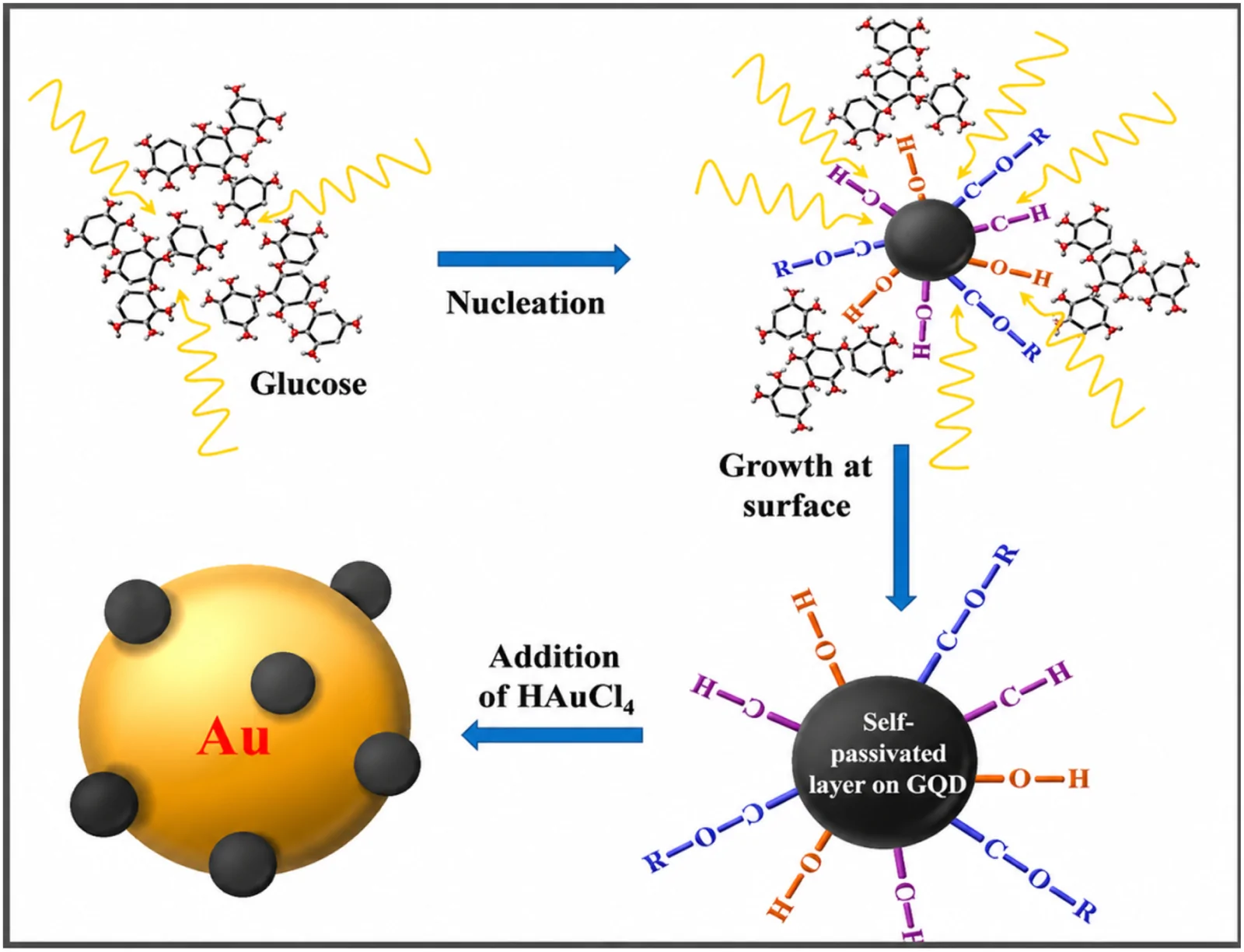
Schematic formation of Au–GQD hybrids through glucose-derived GQD nucleation, surface passivation, and subsequent gold attachment at reactive oxygen-containing sites. Reproduced with permission from ref. 31. © 2020 Elsevier B.V.

### 2.2. Surface chemistry, edge states, and chemical specificity of hybridization

The surface chemistry of GQDs is not a secondary characteristic but a primary determinant of hybrid formation. Because a large fraction of atoms in a GQD are located at edges or near-surface regions, functional groups and defects exert a disproportionate influence on its electronic structure, colloidal behavior, and chemical affinity toward Au. Carboxyl, hydroxyl, carbonyl, amino, thiol, and heteroatom-containing sites can alter local charge density, protonation behavior, coordination capability, and the distribution of occupied and unoccupied states. These groups also determine whether Au species are physically adsorbed, electrostatically associated, coordinatively bound, or covalently anchored.

Different bonding motifs produce fundamentally different interfacial environments. Direct Au–C interaction can provide short electronic coupling distances but may require locally deoxygenated or defect-rich carbon domains. Au–S and Au–N coordination generally provides stronger and more chemically defined anchoring, although the ligand atoms may simultaneously introduce localized states or increase the electronic separation between the metallic and carbon components.^33–35^ Oxygen-containing groups facilitate dispersion and nucleation but may generate heterogeneous binding strengths and unstable coordination under changing chemical conditions. Consequently, a surface optimized for colloidal stability is not necessarily optimized for electronic coupling, and a highly functionalized GQD can exhibit improved processability while suffering from excessive localization or interfacial resistance.

Edge configuration further complicates this relationship. Zigzag- and armchair-like domains, reconstructed edges, vacancies, and sp^3^-rich regions generate different local densities of states and adsorption energies. The apparent surface composition obtained from bulk-averaged characterization therefore does not fully represent the chemically active interface. Two GQD populations with similar elemental composition may interact differently with Au if their functional groups occupy different edge environments or possess different degrees of conjugation with the carbon core.

A rigorous physicochemical description must therefore distinguish between total surface functionality and electronically productive functionality. The relevant parameter is not merely the number of binding groups, but their chemical identity, spatial distribution, accessibility, and electronic communication with the π-framework.^36–38^ This distinction establishes the chemical basis for interfacial coupling and prepares the analysis of how bonding strength, contact distance, and local electrostatics govern the Au–GQD junction.

As shown in Fig. 2, the physicochemical characterization of the Au–GQD nanohybrid highlights the critical role of surface chemistry in governing hybrid formation and interfacial specificity. The UV-visible spectrum (Fig. 2a) confirms the coexistence of GQDs and Au nanoparticles, while the FTIR spectrum (Fig. 2b) identifies oxygen-containing functional groups that provide chemically active sites for Au nucleation and anchoring. The optical analyses (Fig. 2c and d) indicate that hybridization occurs between components with distinct electronic characteristics, whereas the SEM image (Fig. 2e) reveals intimate nanoscale contact that facilitates chemically accessible interfaces. The XRD pattern (Fig. 2f) confirms the coexistence of crystalline Au and graphitic GQD domains following hybrid formation. Collectively, these observations demonstrate that the interfacial environment is governed not only by the presence of functional groups but also by their accessibility, distribution, and chemical interaction with Au, which together determine the nature and effectiveness of Au–GQD hybridization.

**Fig. 2:**
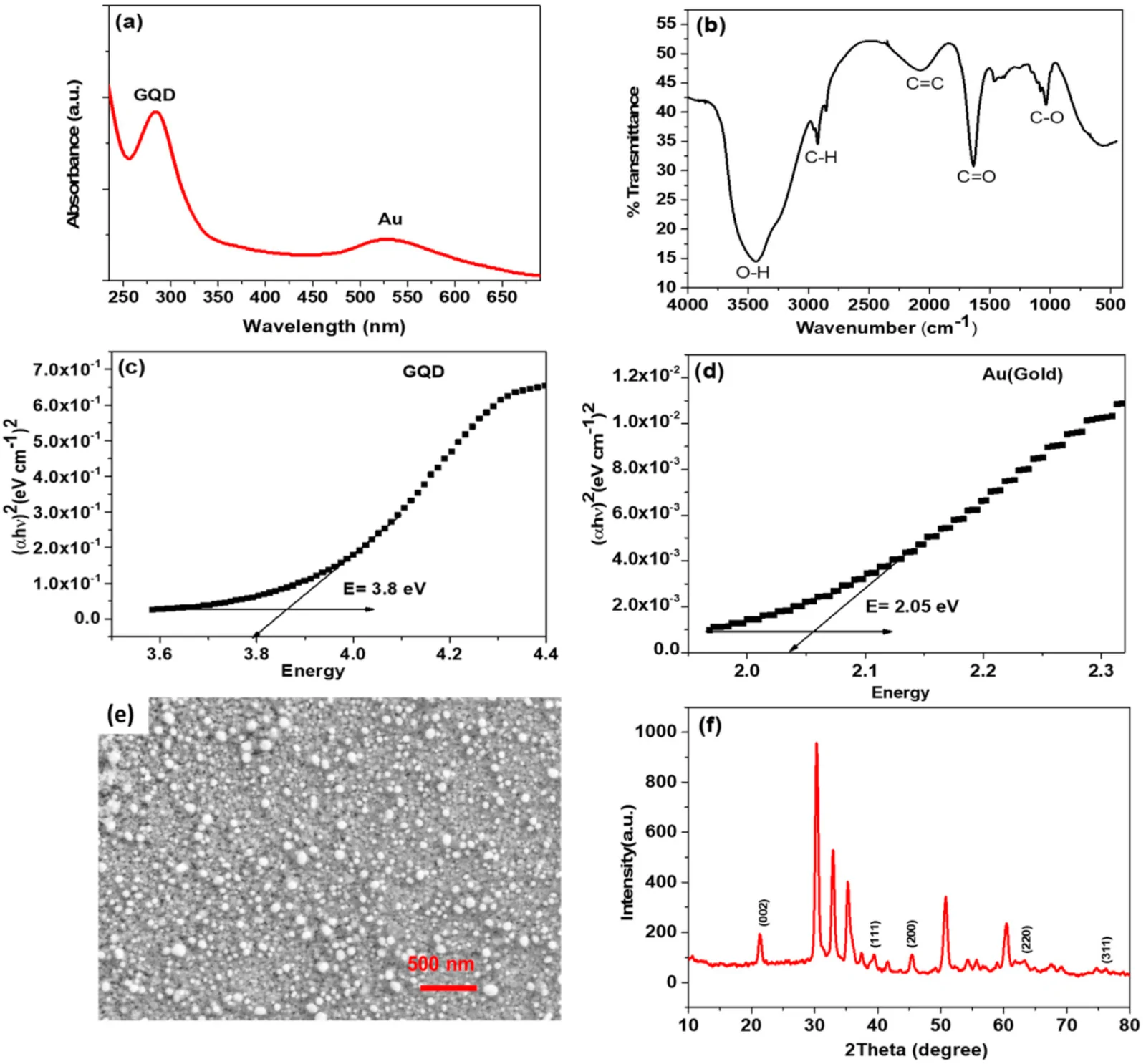
Physicochemical characterization of the Au–GQD nanohybrid: (a) UV-visible absorption spectrum; (b) FTIR spectrum; (c and d) optical band-gap analyses of GQDs and Au; (e) SEM image of the Au–GQD-modified electrode; and (f) XRD pattern of the hybrid. Reproduced from ref. 37 under the Creative Commons Attribution (CC BY) License. © 2024, MDPI.

Quantum-confinement effects arise from the finite dimensions of the conjugated carbon domains, whereas surface-state effects originate from edge configurations, defects, functional groups, and their chemical environments. These contributions can coexist, making the overall particle diameter an insufficient descriptor of the emissive electronic structure. Surface-functionalized Au–GQD architectures demonstrate the importance of characterizing carbon chemistry and hybrid formation alongside optical properties.^31,37^ To distinguish the two contributions, size-dependent measurements should compare GQDs with similar oxidation and functionalization, while surface-state investigations should vary chemical functionality at comparable conjugated-domain dimensions. Optical absorption, emission, and lifetime measurements should be interpreted together with structural and chemical characterization. Excitation-dependent fluorescence or emission quenching alone cannot uniquely identify quantum confinement, surface-localized states, or productive interfacial electron transfer. Where these variables have not been independently controlled, the observed response should be attributed to coupled structural and surface contributions rather than exclusively to quantum confinement.

### 2.3. Interfacial coupling regimes, Fermi-level equilibration, and contact physics

The properties of an Au–GQD hybrid depend not only on whether the two components are connected, but on the coupling regime established at the interface. This regime spans a continuum from weak physisorption to strong chemisorption and partial electronic hybridization. In weakly coupled systems, van der Waals forces or long organic linkers preserve the electronic character of each component but restrict orbital overlap. Charge exchange then proceeds predominantly through tunnelling or thermally assisted transfer across an interfacial barrier. In strongly coupled systems, short metal–carbon distances and chemically defined bonds increase wavefunction overlap, reduce electronic discontinuities, and may create mixed interfacial states.

Fermi-level equilibration provides the thermodynamic foundation of this interaction.^39,40^ When Au and GQDs are brought into contact, their chemical potentials equalize through charge redistribution. This process can generate an interfacial electric field and modify the local vacuum level through dipole formation. However, describing the contact as uniformly Schottky-like or ohmic-like is often an oversimplification. GQDs do not possess a single invariant band edge because their energy levels are distributed across core, edge, defect, and surface-derived states. As a result, the effective barrier can vary spatially within a single hybrid and can be sensitive to adsorbates, protonation, ionic strength, and surface oxidation.

Contact geometry introduces an additional level of complexity. A point contact between a GQD and an Au nanoparticle differs electronically from conformal wrapping, edge attachment, or partial embedding. Increasing the nominal contact area may improve coupling, but it can also create structurally heterogeneous regions with distinct local barriers.^41,42^ Similarly, an intervening ligand layer may stabilize the architecture while suppressing direct electronic communication. Interfacial quality must therefore be assessed through effective coupling strength rather than geometric proximity alone.

The central physicochemical principle is that an Au–GQD junction is controlled by the combined effects of energetic alignment, bond character, contact geometry, dielectric screening, and interfacial disorder. None of these parameters can independently predict the behavior of the hybrid. Their combined influence defines the accessible electronic states and the degree to which the interface behaves as a barrier, a selective contact, or an electronically integrated domain.^43,44^ This coupling framework provides the basis for examining the intrinsic structure–property trade-offs of Au–GQD systems.

As shown in Fig. 3, XPS provides indirect insight into the contact chemistry and electronic redistribution governing the Au–GQD coupling regime. The GQD survey spectrum in Fig. 3a(i) identifies the carbon- and oxygen-rich surface, while the deconvoluted C 1s and O 1s spectra in Fig. 3a(ii) and (iii) resolve sp^2^ carbon, hydroxylated sp^3^ carbon, ether-like C–O–C, carbonyl, and C–O environments that can define the initial interfacial barrier and Au anchoring geometry. After hybridization, the simultaneous detection of C, O, and Au in Fig. 3b(i) and the Au 4f doublet in Fig. 3b(ii) confirm formation of the metallic contact. More importantly, the altered C 1s and O 1s binding-energy distributions in Fig. 3b(iii) and (iv) indicate changes in the local electrostatic environment following Au coupling, consistent with interfacial charge redistribution and dipole formation. These shifts support electronic interaction across the junction, but do not alone establish Fermi-level equilibration, a specific Schottky or ohmic contact, or a unique Au–C/Au–O bonding configuration.

**Fig. 3:**
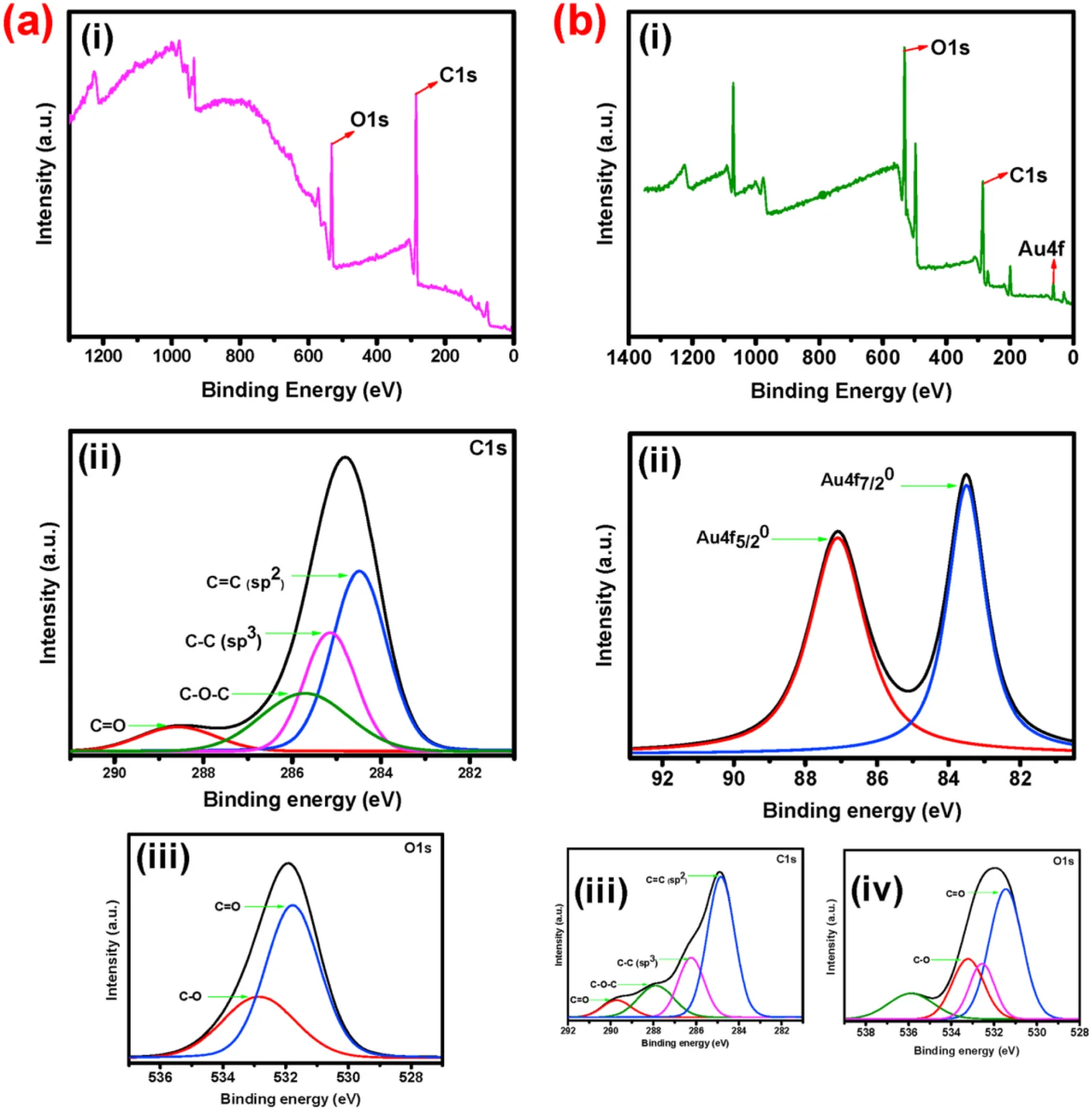
XPS characterization of GQDs and GQDs–GNPs: (a) GQD survey, C 1s, and O 1s spectra; and (b) nanohybrid survey, Au 4f, C 1s, and O 1s spectra, illustrating changes in surface chemical environments after Au coupling. Reproduced with permission from ref. 43. © 2023 Elsevier B.V.

Fermi-level equilibration provides a thermodynamic description of contact formation, but its consequences require experimental verification for Au–GQD architecture. The XPS characterization discussed above establishes the presence of Au and changes in carbon- and oxygen-containing chemical environments after hybridization; these observations support interfacial interaction without independently proving Fermi-level equilibration or a unique contact type.^43^ Stronger assessment requires complementary work-function measurements, contact-potential mapping, and valence-state spectroscopy before and after coupling. Core-level shifts must be interpreted cautiously because coordination changes, differential charging, and final-state screening can also affect binding energies. For heterogeneous GQD ensembles, distributed electronic states further limit the applicability of a uniform bulk-semiconductor band diagram. Dark-state equilibration must also be distinguished from illuminated operation, during which nonequilibrium carrier populations develop. Consequently, a proposed equilibrium alignment should not be presented as direct evidence of photoinduced transfer direction unless supported by independent carrier-sensitive measurements.

### 2.4. Structure–property relationships, intrinsic trade-offs, and conceptual gaps

The structure–property relationship in Au–GQD hybrids is governed by competing requirements rather than a single optimization variable. Smaller GQDs can provide stronger quantum confinement and a greater fraction of edge sites, yet they also exhibit higher structural heterogeneity and stronger sensitivity to surface oxidation. Larger conjugated domains may support greater electronic delocalization but reduce the relative contribution of chemically addressable edges. Similarly, decreasing the size of Au nanoparticles increases the proportion of low-coordination atoms and alters the electronic density of states, while very small clusters may no longer exhibit the collective metallic behavior associated with larger plasmonic particles.

Surface functionalization introduces another intrinsic trade-off. High functional-group density can improve dispersion, chemical anchoring, and control over Au nucleation. At the same time, excessive oxidation or sp^3^ disruption can interrupt the conjugated carbon framework and increase localization. Strong interfacial bonding can reduce contact resistance, but it may also perturb the electronic states responsible for light absorption or create deep traps.^45–47^ Conversely, preserving the intrinsic states of both components through weak coupling can maintain their separate optical identities while limiting electronic communication. The desired interface is therefore not necessarily the strongest possible interface, but one that balances structural stability with controlled electronic interaction.

A major conceptual gap is the absence of universally transferable descriptors. Particle size, average GQD diameter, elemental composition, and nominal Au loading are insufficient because they do not resolve bonding configuration, contact geometry, local work function, edge distribution, or the density of electronically active states. The field also frequently treats GQDs as compositionally uniform semiconductors, although they are better understood as ensembles of chemically and electronically heterogeneous nanodomains. Similarly, Au is often represented as a passive metallic component despite its size-dependent surface electronic structure and sensitivity to ligands and adsorbates.

A more rigorous framework should link measurable structural descriptors to electronic consequences. Relevant descriptors include the distribution of GQD energy states, fraction of conjugated carbon, interfacial bond identity, Au coordination environment, contact distance, work-function difference, and spatial heterogeneity of the interface.^48–50^ Establishing these relationships is essential before higher-level transport and reaction phenomena can be interpreted. Thus, Section 2 defines the material-level basis of Au–GQD hybridization, while the following section can examine how these foundational properties govern the multiscale movement of photons, excitations, charges, and reactants.

As summarized in Table 1, the physicochemical behavior of Au–GQD hybrids emerges from the combined influence of electronic dimensionality, surface-state distribution, bonding configuration, contact geometry, and local electrostatics. The table highlights that commonly reported descriptors such as average particle size, elemental composition, or Au loading are insufficient when considered independently. Instead, predictive interpretation requires coupling structural descriptors with their electronic consequences and recognizing intrinsic trade-offs between interfacial stability, orbital communication, and energetic selectivity. This framework provides a rigorous basis for analyzing subsequent excitation, charge-transfer, and redox processes.

**Table 1:** Conceptual matrix of physicochemical descriptors governing Au–GQD hybridization and interfacial properties

Foundational descriptor	Physicochemical basis	Mechanistic role at the interface	Structure–property consequence	Intrinsic limitation or trade-off	Design significance
Au electronic dimensionality	The density of electronic states evolves from quasi-continuous metallic bands to discrete cluster-like levels as Au dimensions decrease	Determines carrier availability, Fermi-level position, surface coordination, and sensitivity to adsorbates	Controls whether Au retains collective metallic character or exhibits molecule-like electronic behavior	Smaller dimensions increase low-coordination sites but may suppress the electronic characteristics associated with larger metallic domains	Au size should be selected according to the required electronic regime rather than minimized indiscriminately
GQD π-domain size	Finite lateral dimensions and quantum confinement regulate the separation and distribution of occupied and unoccupied states	Defines the energetic accessibility and spatial delocalization of confined electronic states	Smaller conjugated domains strengthen confinement and increase edge contribution; larger domains favor delocalization	Strong confinement can increase energetic tunability but also amplify structural and chemical heterogeneity	Size distribution must be considered alongside conjugation length and edge-to-core ratio
Edge and defect states	Zigzag-like edges, armchair-like edges, vacancies, reconstructed sites, and sp^3^ defects generate non-equivalent localized states	Provide electronically accessible sites for interaction with Au and modify the local density of states	Can introduce tunable interfacial states while altering localization, orbital continuity, and chemical affinity	Excessive or poorly defined defects may generate deep states and obscure intrinsic quantum-confinement effects	Defect identity and spatial distribution are more informative than total defect density alone
Surface functional groups	Carboxyl, hydroxyl, carbonyl, amino, and thiol groups modify charge density, protonation, coordination, and solvation	Regulate Au nucleation, anchoring strength, interfacial distance, and local electrostatics	Improve chemical addressability and colloidal stability but may disrupt the conjugated carbon framework	High functionalization can enhance processability while increasing electronic localization and interfacial resistance	Surface chemistry should balance chemical accessibility with electronic communication to the GQD core
Heteroatom configuration	Nitrogen-, sulfur-, and other heteroatom-containing sites perturb local orbital energies and coordination environments	Create chemically selective binding sites and modify the work function and local charge distribution	Enables stronger or more defined Au coordination and introduces additional electronic-state heterogeneity	Nominal dopant concentration does not reveal bonding configuration, accessibility, or electronic activity	Dopant bonding state and location should be controlled rather than relying on bulk elemental content
Work-function mismatch	Au and GQDs possess distinct chemical potentials before contact, both of which depend on size and surface condition	Drives charge redistribution until electrochemical equilibrium is established	Produces shifts in effective Fermi level and alters the energetic character of the junction	GQD work function is not invariant and can change with oxidation, protonation, adsorbates, and structural disorder	Work-function differences must be measured under chemically relevant conditions rather than inferred from idealized values
Interfacial dipole formation	Redistribution of charge and asymmetric bonding modify the local vacuum level at the Au–GQD boundary	Generates local electrostatic fields and changes the effective energetic offset between the components	Can transform an apparently neutral contact into an electronically selective or resistive junction	Dipole magnitude and direction may vary spatially because of nonuniform functionalization and adsorption	Local interfacial electrostatics should be treated as an independent descriptor of hybrid identity
Interfacial bonding motif	Au–C, Au–S, Au–N, oxygen-mediated coordination, and noncovalent association provide different orbital overlaps	Governs anchoring stability, electronic coupling strength, and the degree of orbital hybridization	Direct bonding can shorten the coupling distance, whereas ligand-mediated contacts may preserve component-specific states	Strong bonding may perturb desirable electronic states; weak bonding may preserve them but restrict electronic communication	The optimum interface is electronically controlled rather than simply the most strongly bonded configuration
Contact geometry and distance	Point attachment, edge binding, conformal wrapping, partial embedding, and linker-separated contacts create distinct boundaries	Determines effective contact area, tunnelling distance, orbital overlap, and spatial barrier distribution	Larger nominal contact areas may strengthen coupling but can also increase interfacial heterogeneity	Geometric proximity does not necessarily correspond to low electronic resistance or uniform coupling	Contact quality should be assessed through effective electronic coupling, not microscopy-derived proximity alone
Coupling regime	Hybridization spans weak physisorption, intermediate coordination, strong chemisorption, and partial orbital mixing	Determines whether Au and GQDs retain separate electronic identities or form mixed interfacial states	Weak coupling preserves component-specific properties; strong coupling broadens states and reconstructs the interface	Either extreme may be unfavorable because weak coupling limits communication, while excessive mixing can eliminate functional state selectivity	Coupling strength should be optimized as a continuum rather than classified only as “coupled” or “uncoupled.”
Interfacial heterogeneity and environmental sensitivity	Variations in size, edge structure, bond identity, dielectric screening, adsorbates, and ionic environment create distributed local interfaces	Produce spatially variable barriers, work functions, coupling strengths, and electronic-state populations	Causes ensemble-averaged measurements to conceal minority interfaces that may dominate hybrid behavior	Average size, composition, and loading cannot provide transferable structure–property relationships	Descriptor sets should integrate energy-state distributions, bond identity, coordination, contact geometry, and local environment

## 3. Coupled-flux regulation in solar-to-chemical energy conversion: from photon capture to interfacial redox turnover

### 3.1. Spectral utilization and photon-flux management

Solar-to-chemical conversion is often discussed in terms of light absorption, yet total absorbance alone is an incomplete descriptor of functional performance. The decisive quantity is the fraction of incident photons that is absorbed within an energetically useful spectral window, at a spatial location connected to subsequent charge-generation and reaction pathways. Optical design must therefore regulate not only the magnitude of absorption but also its wavelength dependence, penetration depth, spatial distribution, and compatibility with the excitation thresholds of the photoactive system.

A central limitation arises from the mismatch between the broad solar spectrum and the narrower absorption profiles of individual materials. Expanding spectral coverage can increase photon capture, but it may simultaneously introduce sub-bandgap absorption, parasitic heating, or excitation into states that cannot support the required redox chemistry.^51–53^ Optical intensification is therefore beneficial only when it increases the population of electronically productive states. Absorption associated with thermally dissipative channels may improve apparent light harvesting while contributing little to chemical conversion.

Photon transport within the reactor or electrode architecture also imposes competing constraints. Thick photoactive layers improve the probability of absorption but lengthen the distance over which generated carriers must travel. Thin layers reduce transport losses but may transmit a substantial fraction of incident light. Likewise, high optical density near the illuminated surface can create steep excitation gradients, leaving deeper regions underutilized. Scattering and internal reflection may partially redistribute photon flux, although excessive scattering can increase optical losses or produce nonuniform excitation fields.

The appropriate design objective is therefore not maximum absorbance, but a controlled balance between spectral coverage, absorption depth, and spatial accessibility of the generated excitation. Photon-management strategies should be evaluated through an action-spectrum perspective in which optical absorption is weighted by its probability of producing chemically useful charge. This establishes a distinction between incident, absorbed, and productively utilized photon flux.^54–56^ Once this distinction is made, the analytical focus must shift from photon interception to the fate of the excited states created by absorption.

### 3.2. Excited-state partitioning and productive charge generation

Absorbed photons generate a heterogeneous population of excited states that may evolve through charge separation, energy transfer, trapping, radiative relaxation, or thermal dissipation. The efficiency of solar-to-chemical conversion therefore depends on how this initial population is partitioned among competing pathways. Treating all photoexcited states as equivalent obscures the fact that only a subset possesses the lifetime, energy, and spatial character required to generate redox-active carriers.

The transition from optical excitation to productive charge generation is governed by kinetic competition. Excited-state relaxation may occur on a timescale shorter than interfacial separation, particularly when strong electron–phonon coupling, structural disorder, or dense nonradiative states are present. Conversely, rapid charge separation can preserve a greater fraction of excitation energy, but only if the resulting electron and hole remain sufficiently energetic to drive the intended reactions.^57–59^ A pathway that efficiently separates charge after substantial energetic relaxation may generate long-lived carriers with inadequate redox potential. Thus, charge-separation yield and retained free energy must be considered jointly.

Excited-state localization introduces an additional design tension. Delocalized states generally support rapid carrier transport, whereas localized states can reduce wavefunction overlap and suppress recombination. However, excessive localization may immobilize charge or isolate it from reactive surfaces. Trap states are therefore neither inherently beneficial nor uniformly detrimental. Shallow, reversible trapping can extend carrier lifetime and facilitate directional migration, while deep or chemically unstable traps may act as terminal loss sites. The functional distinction depends on trap depth, occupancy, detrapping kinetics, and proximity to reaction sites.

A useful analytical framework should separate three sequential probabilities: formation of an energetically competent excitation, conversion of that excitation into a spatially separated carrier pair, and survival of the pair long enough to enter transport pathways^60–62^ Improving one probability does not guarantee improvement in the others. The governing design principle is to maximize the yield of redox-competent separated charges rather than the lifetime or intensity of any single excited-state signature. This requirement leads directly to the problem of how generated carriers are transported and selectively delivered.

As illustrated in Fig. 4, the thermodynamic landscape of artificial photosynthesis clarifies why photon absorption alone is insufficient for solar-to-chemical conversion. The direct conversion of CO_2_ and H_2_O into reduced carbon products and O_2_ is an overall endergonic process, requiring photoexcited electrons and holes to retain sufficient free energy after excitation, relaxation, and charge separation. The schematic further resolves this transformation into two energetically distinct stages: an uphill water-splitting step that generates H_2_ and O_2_, followed by CO_2_ hydrogenation, which is thermodynamically more favorable for many reaction pathways. This energetic separation highlights the central role of excited-state partitioning, because only carriers that are successfully separated while preserving adequate reduction and oxidation potentials can contribute to productive chemical conversion. Carriers lost through radiative decay, nonradiative relaxation, or deep trapping do not contribute to the required thermodynamic work, even when light absorption is strong. Fig. 4 therefore emphasizes that productive charge generation must be evaluated through both separation yield and retained redox energy, while also showing how renewable H_2_ can function as an intermediate chemical-energy carrier linking solar-driven water splitting to downstream CO_2_ conversion.

**Fig. 4:**
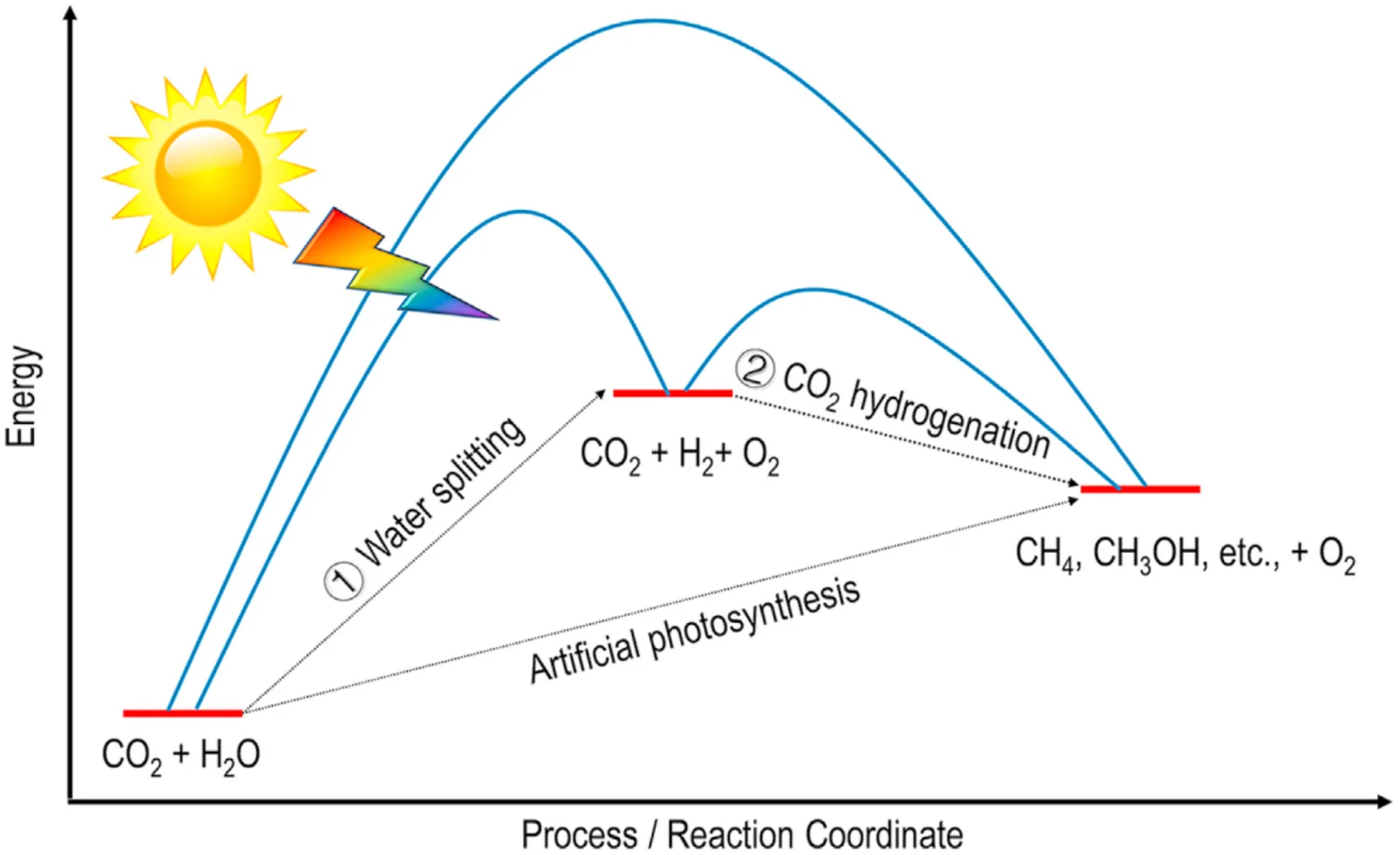
Solar-driven water splitting and subsequent CO_2_ hydrogenation. Reproduced from ref. 61 under the Creative Commons Attribution (CC BY) License. © 2022 Cell.

### 3.3. Carrier transport, selective extraction, and recombination control

After charge generation, the system must route electrons and holes toward spatially distinct reaction environments while minimizing bulk, surface, and interfacial recombination. Carrier transport is therefore not merely a question of mobility; it is a problem of directional selectivity, transit time, energetic retention, and synchronization with surface consumption. A material with high intrinsic conductivity may still perform poorly if charge motion is nondirectional or if carriers reach nonproductive interfaces.

The effective transport distance must remain compatible with the carrier diffusion length and lifetime. Increasing crystallinity or electronic connectivity may reduce transport resistance, but it can also decrease the density of sites that promote charge separation or surface adsorption. Conversely, defect-rich structures may enhance localization and interfacial reactivity while introducing scattering, trapping, and mobility loss.^63–65^ The optimum transport network is therefore neither maximally ordered nor maximally defective, but sufficiently connected to support carrier movement without eliminating the energetic asymmetry required for directional extraction.

Selective contacts and internal electric fields can bias carrier flow, yet their effectiveness depends on barrier height and kinetic asymmetry. A barrier that suppresses reverse transfer may also impede forward transport if it is too large. Similarly, a low-resistance junction can accelerate charge extraction while allowing rapid back-transfer when surface reactions are slow. Recombination control must consequently be linked to reaction kinetics rather than evaluated solely through prolonged carrier lifetime. Long-lived carriers are beneficial only when they remain mobile, retain adequate potential, and can be consumed by interfacial reactions.

Charge accumulation further alters the transport landscape. When one carrier type is extracted or consumed more slowly, local electrostatic potentials shift, transport barriers change, and recombination pathways may become more favorable. This dynamic feedback means that carrier routing cannot be treated as a static property measured independently of operating conditions. The transferable design principle is to match separation rate, transport time, extraction selectivity, and surface-consumption rate within the same kinetic window.^66–68^ Carrier transport becomes productive only when it delivers redox-capable charges to accessible sites faster than recombination removes them. This establishes the need to examine the molecular and interfacial processes that ultimately consume those carriers.

As illustrated in Fig. 5, integrated thin-film architectures regulate carrier transport by combining spatially separated reaction sites with selective conductive pathways. In the tandem photoelectrochemical device shown in Fig. 5a, the BiVO_4_ photoanode and encapsulated perovskite photocathode generate and direct holes and electrons toward oxygen evolution and CO_2_ reduction, respectively, while the electron- and hole-transport layers and conductive encapsulation promote selective extraction and suppress nonproductive carrier loss. The single-absorber artificial leaf in Fig. 5b similarly couples a perovskite light absorber to spatially distinct Pt and Cu–Pd electrocatalysts, enabling photogenerated electrons to drive proton reduction while holes are consumed through organic oxidation. In the Z-scheme photocatalyst sheet presented in Fig. 5c, doped BiVO_4_ and SrTiO_3_ particles are electronically connected through a solid Au layer that functions as an interparticle charge relay, facilitating selective recombination of low-energy carriers while preserving the strongly reducing and oxidizing carriers required for fuel formation. The immobilized CN_*x*_-based sheet in Fig. 5d integrates semiconductor particles, conductive support, and reduction cocatalysts within a compact architecture, shortening carrier-transport distances and coupling hydrogen evolution with organic-substrate oxidation. Collectively, these configurations demonstrate that effective recombination control depends not only on charge-carrier lifetime, but also on directional extraction, contact selectivity, transport-path continuity, and sufficiently rapid carrier consumption at spatially accessible catalytic sites.

**Fig. 5:**
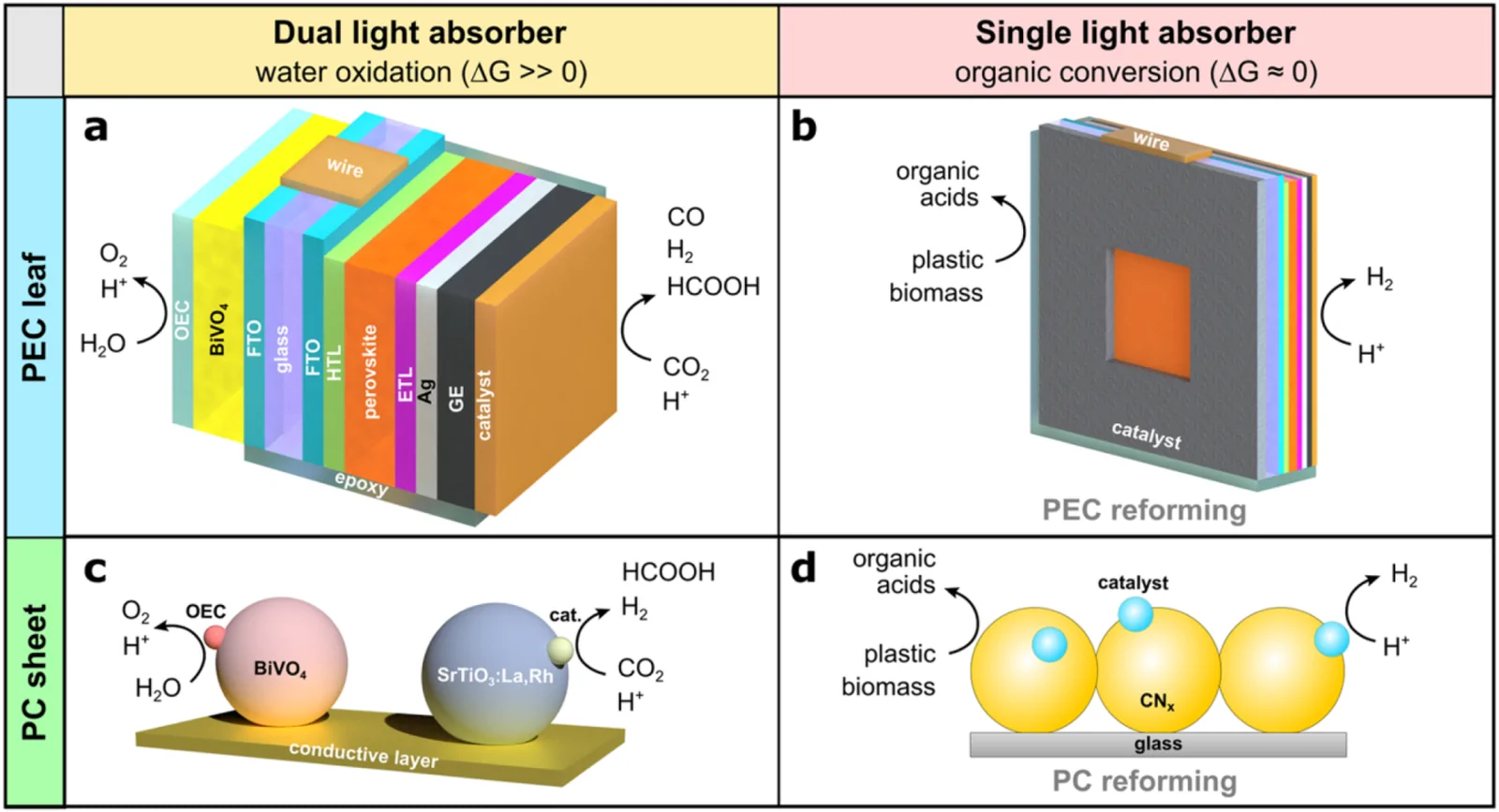
Thin-film solar-fuel architectures for directional carrier transport and selective extraction: (a) tandem BiVO_4_–perovskite artificial leaf; (b) perovskite device coupling proton reduction with organic oxidation; (c) Au-mediated Z-scheme photocatalyst sheet; and (d) immobilized semiconductor sheet for H_2_ evolution and organic reforming. Reproduced with permission from ref. 67. © 2022 American Chemical Society.

### 3.4. Reactant delivery, redox-pathway synchronization, and product removal

The final stage of solar-to-chemical conversion is governed by the interaction between electronic flux and molecular flux. Even when photon utilization, charge generation, and carrier transport are favorable, conversion can remain limited by slow reactant diffusion, restricted active-site accessibility, proton-transfer resistance, or product accumulation. The reaction interface must therefore be understood as a dynamic zone in which charge, mass, and chemical intermediates are continuously exchanged. Reactant delivery depends on porosity, wettability, surface charge, solvation structure, and local concentration gradients. Increasing surface area may raise the nominal number of reaction sites, but it does not guarantee that these sites are accessible under operating conditions. Narrow pores can hinder diffusion, surface ligands can block adsorption, and nonuniform wetting can isolate parts of the catalyst from the electrolyte.^69,70^ A chemically active surface may consequently remain underutilized if molecular transport is not coordinated with carrier arrival.

The reduction and oxidation half-reactions also differ in kinetic complexity. Proton reduction may proceed through a limited number of adsorbed intermediates, whereas water oxidation generally requires sequential charge accumulation and multiple bond-forming steps. This asymmetry can create a bottleneck in one half-reaction, causing the opposite carrier to accumulate and increasing recombination. Efficient operation therefore requires redox synchronization rather than isolated optimization of a single half-reaction. Intermediate binding introduces a further trade-off. Weak adsorption limits surface activation, whereas excessively strong adsorption blocks active sites and impedes product desorption.

The appropriate binding strength must support intermediate formation without stabilizing reaction products or partially transformed species. Product removal is equally important because retained gas bubbles, dissolved products, or locally altered pH can reduce active area, modify optical conditions, and redirect selectivity. The central framework that emerges is one of coupled-flux continuity.^71,72^ Photon absorption, excited-state generation, carrier transport, reactant supply, interfacial conversion, and product evacuation must operate at mutually compatible rates. A gain at one stage has limited value when a downstream bottleneck remains dominant. This process-level framework provides a basis for evaluating experimental evidence in the following section without conflating material fundamentals with reported performance.

## 4. Evidence-based analysis of Au–GQD hybrid systems for solar energy conversion

The evidence base for Au–GQD systems in solar-driven energy conversion remains limited but mechanistically informative. Four studies directly address hydrogen generation, water splitting, or photoelectrochemical conversion, whereas four additional contributions provide supporting insight into hybrid formation, plasmonic absorption, hot-electron transfer, and surface-state-dependent interfacial interactions. The direct studies establish the principal functional roles of Au–GQD coupling in solar-fuel pathways, including sensitization, charge extraction, catalytic hydrogen evolution, and suppression of recombination. By contrast, the supporting studies do not demonstrate solar-fuel production and should therefore be interpreted as mechanistic or materials-level evidence rather than as performance benchmarks. Considered together, these reports reveal that the field is defined less by the number of available systems than by the diversity of interfacial functions assigned to Au and GQDs. This section therefore evaluates the literature through synthesis–structure–property–performance relationships, while distinguishing direct conversion evidence from indirect optical and electronic support.

### 4.1. Hybrid formation, structural accessibility, and optical activation

The synthesis route reported for GQD–Au hybrid nanoparticles establishes that interfacial integration can be achieved under mild conditions without complex processing. GQDs simultaneously acted as reducing and supporting components during the conversion of the Au precursor, allowing hybrid formation at room temperature within approximately five minutes. The resulting structures covered a relatively broad size range of 5–100 nm and remained stable for up to four weeks after citrate-assisted extraction.^73^ Although this study did not examine solar-energy conversion, it identifies an important structure–processing relationship: direct precursor reduction by GQDs can simplify hybrid formation and reduce the need for external reducing agents, but the broad particle-size distribution may produce nonuniform optical and electronic interfaces.

The optical consequences of forming a core–shell-like Au@GQD architecture were examined through broadband photodetection. The Au core extended spectral absorption, while the surrounding GQD-derived carbon layer contributed to strong fluorescence quenching and electronic communication. A photoresponsivity of approximately 10^3^ A W^−1^, detectivity of 10^13^ Jones, and millisecond-scale response were obtained.^74^ These values cannot be transferred directly to solar-fuel conversion because photodetection measures electrical signal generation rather than chemical-energy storage. Nevertheless, they confirm that intimate Au–GQD contact can broaden photon utilization and promote rapid excited-state processing.

A related optical study showed that Au concentration alters the nature of plasmon–GQD interaction. At lower Au concentrations, fluorescence increased by 2.67-fold, whereas higher concentrations generated selective excitation features attributed to energetic-electron transfer from Au to GQDs.^75^ The transition from field-enhanced emission to carrier-transfer-dominated behavior indicates that Au loading is not a monotonic performance variable. Excess metal may modify spectral response but can also introduce quenching or dissipative pathways.

Collectively, these studies suggest that rapid hybrid formation and strong optical coupling are achievable, but neither guarantees solar-fuel activity. Particle-size uniformity, Au loading and contact configuration must be controlled because they determine whether absorbed light produces useful interfacial carriers or predominantly optical enhancement.^73,76^

As shown in Fig. 6, the fluorescence response of AuNPs/G6/GQDs composites is strongly governed by the surface-state chemistry of the GQDs rather than by Au incorporation alone. For low-oxidation GQDs1, the spectra in Fig. 6A show pronounced fluorescence quenching after coupling with AuNPs and G6, indicating efficient interfacial energy transfer enabled by exposed hydroxyl-rich sites and close Au–GQD association. At an intermediate oxidation level, Fig. 6B shows only weak quenching for AuNPs/GQDs2 and slight fluorescence recovery after insertion of the G6 linker, consistent with increased carboxyl coverage weakening direct interaction with hydroxyl groups. For highly oxidized GQDs3, Fig. 6C reveals renewed fluorescence loss, likely reflecting both altered interfacial binding and oxidation-induced restructuring of the GQD emissive states. The normalized changes summarized in Fig. 6D therefore demonstrate that surface oxidation degree and linker-controlled spacing jointly determine whether Au–GQD coupling promotes quenching, recovery, or structural perturbation. These results support the conclusion that optical activation in Au–GQD hybrids depends critically on the chemical accessibility and distribution of surface states.

**Fig. 6:**
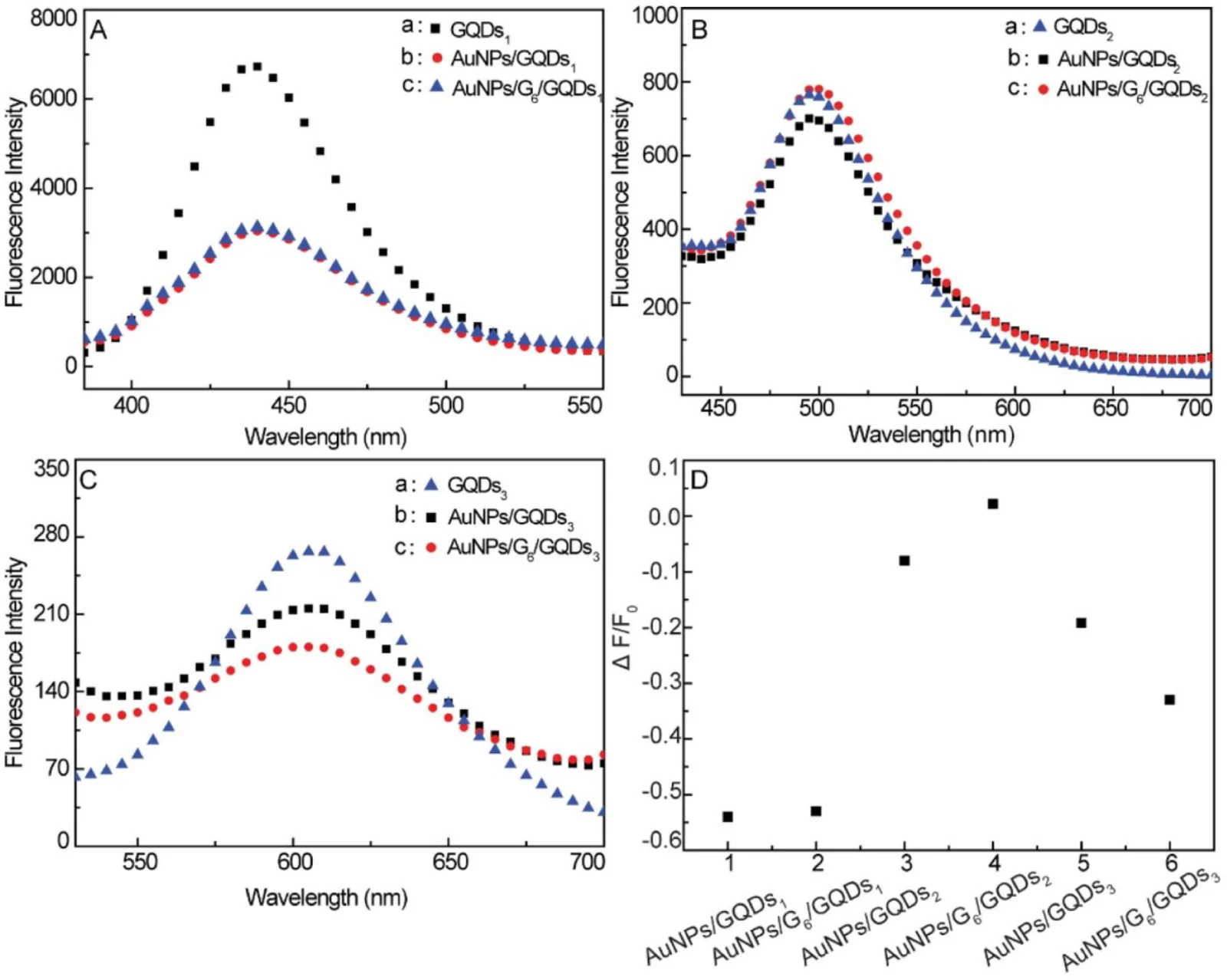
Surface-state-dependent fluorescence of AuNPs/G6/GQDs composites: (A–C) spectra for differently oxidized GQDs and (D) normalized intensity changes. Reproduced with permission from ref. 76. © 2021 Elsevier B.V.

### 4.2. Interfacial energy-level alignment and directional electron transfer

The clearest evidence for directional charge transfer is provided by Au nanocluster–GQD composites in which the energetic levels form a type-II alignment. Au nanoclusters functioned as visible-light sensitizers and electron donors, while GQDs acted as electron-accepting domains. Fluorescence quenching and shortened Au-cluster lifetimes were consistent with electron transfer from Au to GQDs, and the composite photocurrent was 2.69 and 4.80 times that of the isolated Au nanoclusters and GQDs, respectively.^77^ Unlike optical enhancement alone, this result links energetic alignment to a measurable increase in separated-carrier generation.

However, the type-II description should not be interpreted as universally applicable to every Au–GQD interface. Surface chemistry modifies the electronic states of GQDs, and the contribution of those states was directly demonstrated in composites containing GQDs with different surface configurations. Changes in GQD surface state altered photoexcited energy-transfer efficiency and the resulting spectroscopic response, even when quantum-size effects were excluded.^76^ This finding indicates that nominally similar GQDs may produce different transfer pathways because surface-state distributions change the energetic and spatial character of the accepting levels.

The two studies together reveal a central structure–property relationship. Energy-level alignment determines the thermodynamic direction of transfer, whereas surface-state character influences the kinetic probability and final destination of the transferred excitation or carrier.^70,77^ A favorable nominal alignment may therefore remain ineffective if electrons are captured by deep or poorly connected states. Conversely, surface states can improve interfacial transfer when they act as accessible intermediate levels without becoming terminal recombination centres.

Evidence from plasmon-enhanced fluorescence provides further support for concentration-dependent carrier injection. The proposed transfer of energetic electrons from Au nanoparticles to GQDs at higher Au concentration is consistent with an interface that changes from predominantly electromagnetic coupling to direct electronic exchange.^68,75^ Yet fluorescence enhancement or quenching alone cannot establish the fraction of carriers available for chemical conversion.

Accordingly, the available evidence supports directional Au-to-GQD electron transfer, but it also exposes a characterization gap. Band alignment, lifetime changes, fluorescence response, and photocurrent should be interpreted jointly. No single descriptor is sufficient to distinguish productive charge separation from energy transfer, trapping, or nonradiative relaxation.^76^ The three-electrode configuration in Fig. 7a was then used to evaluate the photoelectric response, while the LSV curves in Fig. 7b, transient photocurrent in Fig. 7c, and EIS spectra in Fig. 7d demonstrate enhanced carrier separation, higher photocurrent generation, and reduced interfacial resistance in the hybrid. Together, these results link type-II energy-level alignment and ultrafast Au-to-GQD electron transfer to improved charge extraction and photoelectric performance.

**Fig. 7:**
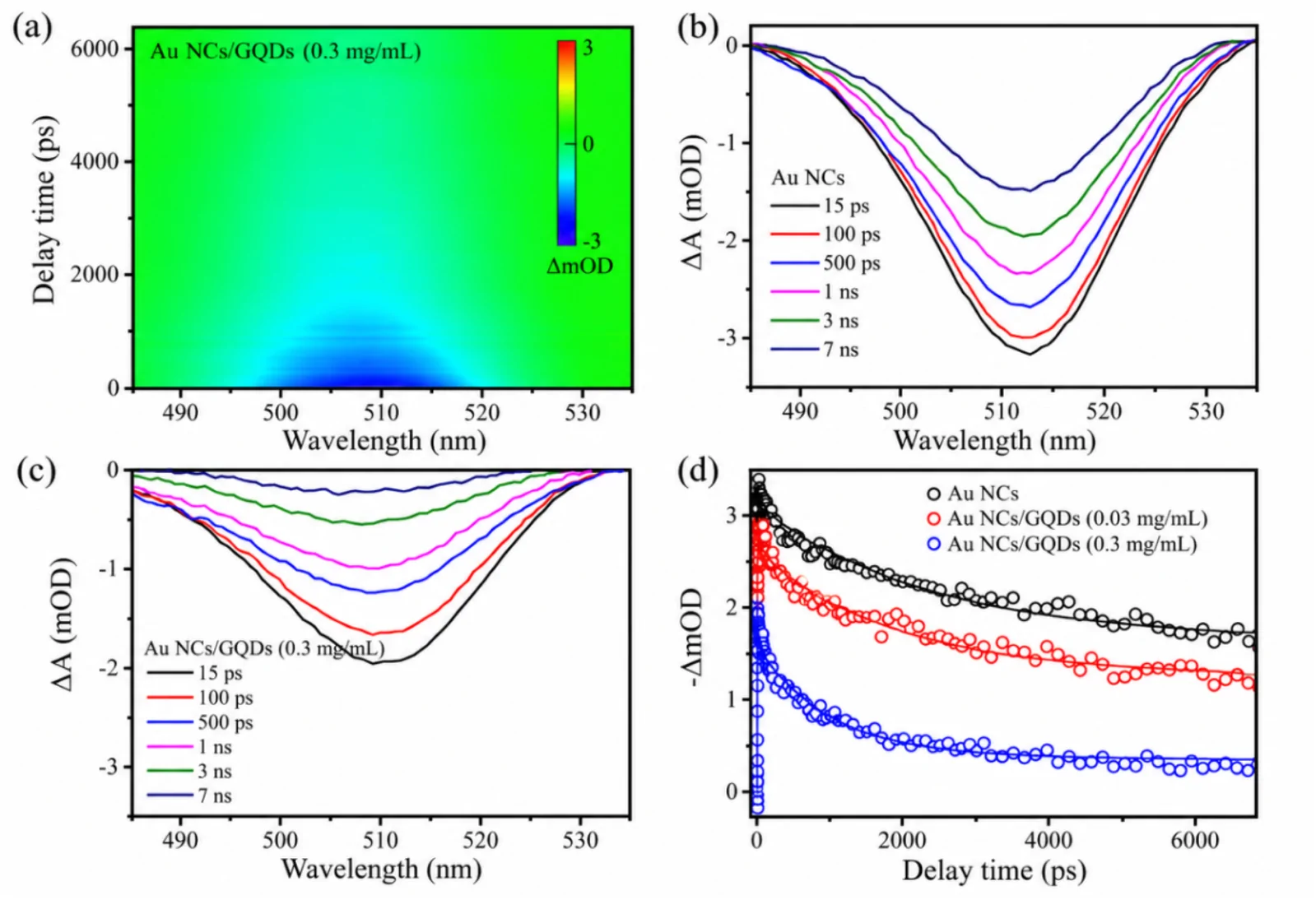
Photoelectrochemical properties of Au NCs, GQDs, and their composite: (a) cell configuration, (b) LSV, (c) photocurrent, and (d) EIS. Reproduced with permission from ref. 77. © 2023 Elsevier B.V.

Evidence for Au-to-GQD electron transfer should be distinguished from fluorescence quenching alone. Among the studies considered here, ref. 77 provides the clearest convergent support: Au-nanocluster emission quenching and lifetime shortening are accompanied by reported type-II energetic alignment, agreement between steady-state and dynamical transfer estimates, and enhanced photocurrent. Together, these observations support the proposed donor–acceptor pathway more strongly than emission-intensity changes alone. Nevertheless, lifetime shortening identifies an additional excited-state decay channel and requires complementary evidence to distinguish electron transfer from other nonradiative processes. Furthermore, electron donation by molecular-like Au nanoclusters should not automatically be equated with plasmon-induced hot-electron injection from metallic nanoparticles. The related N-rGO/NiFe-LDH@Au photoelectrode illustrates plasmon-assisted interfacial transport in a different carbon-containing architecture.^93^ Its relevance is comparative: reduced graphene oxide is not GQD, and enhanced photoelectrochemical performance cannot independently establish an Au-to-GQD transfer direction.

### 4.3. Hydrogen production and overall water-splitting pathways

Direct hydrogen-production evidence is provided by Au deposited on sulfur-doped GQDs for the visible-light-driven degradation of formic acid. Thiol-containing sites facilitated Au coordination, creating a chemically anchored interface that supported hydrogen generation with a turnover frequency of up to 112 h^−1^.^78^ This system demonstrates that heteroatom-mediated Au attachment can combine structural stabilization with catalytic accessibility. However, the process uses formic acid as the hydrogen source and therefore represents photocatalytic dehydrogenation rather than unbiased water splitting. Its relevance to solar-fuel generation lies mainly in showing how chemically defined Au–GQD contacts can mediate light-assisted hydrogen evolution under mild conditions.

A broader reaction pathway was reported for simultaneous production of H_2_ and H_2_O_2_ from water. Nitrogen-doped GQDs underwent bottom-up polycondensation into larger graphene domains during Au-assisted processing, yielding graphene-wrapped Au structures. The resulting catalyst produced H_2_ and H_2_O_2_ at rates of 65.6 and 49.7 µmol g^−1^ h^−1^, respectively.^79^ Simultaneous formation of these products avoids the conventional H_2_/O_2_ combination and introduces H_2_O_2_ as a value-added oxidation product. Nevertheless, the structural transformation of GQDs into graphene means that the final catalyst is not a conventional Au–GQD hybrid. It should therefore be treated as evidence of GQD-enabled architectural evolution rather than as a direct benchmark for stable Au–GQD interfaces.

Comparison of the two studies highlights distinct functions of GQDs. In the formic-acid system, sulfur-doped GQDs serve as Au-binding and electronically active supports. In overall water splitting, N-GQDs act partly as precursors for constructing a graphene shell around Au. Thus, the term “GQD contribution” covers both persistent quantum-dot functionality and sacrificial structural transformation.

The evidence also remains difficult to compare quantitatively because the studies report different reactions, products, and activity metrics. Turnover frequency in formic-acid conversion cannot be directly ranked against mass-normalized H_2_ and H_2_O_2_ production rates.^58,60^ The literature therefore establishes chemical feasibility but does not yet provide a standardized basis for determining whether quantum confinement, dopant chemistry, Au catalysis, or structural reconstruction is the dominant source of activity.

### 4.4. Photoelectrochemical water splitting and cross-study design implications

The most direct evidence for photoelectrochemical water splitting is provided by a TiO_2_-based photoelectrode containing Au nanoparticles and grafted GQDs. The hybrid containing larger Au particles delivered a current density of 0.535 mA cm^−2^ at 1.23 V *versus* RHE, compared with 0.267 mA cm^−2^ for pristine TiO_2_.^80^ This approximately twofold increase was attributed to the combined influence of localized surface plasmon resonance and GQD-mediated extraction of energetic electrons from Au, which reduced charge recombination. In contrast, smaller Au nanoparticles impeded electron transport because of their greater electron affinity. The result directly demonstrates that reducing Au size does not necessarily improve conversion; particle size must be matched to the required balance between plasmon generation and carrier release.

This size-dependent behavior complements the type-II Au-nanocluster/GQD system, where efficient electron donation from Au clusters to GQDs enhanced photocurrent.^77^ Together, the studies show that the role of Au depends on its electronic regime. Molecular-scale clusters can act as sensitizing electron donors when their energy levels align appropriately, whereas larger plasmonic particles can generate energetic carriers through collective excitation. Intermediate or excessively electron-affine structures may instead retain charge and impede transfer.

The hydrogen-evolution and overall water-splitting studies add a chemical dimension to this trend. Strong Au–S coordination supported visible-light hydrogen generation,^78^ while transformation of N-GQDs into graphene around Au produced a structurally integrated catalyst for simultaneous H_2_/H_2_O_2_ formation.^79^ Supporting optical studies further indicate that Au concentration, hybrid geometry, and GQD surface states govern whether coupling produces field enhancement, carrier injection, or energy dissipation.^74^

Table 2 reveals that the strongest evidence for interfacial charge dynamics and the strongest evidence for chemical conversion arise from different experimental systems. The Au-nanocluster/GQD composite combines steady-state and time-resolved measurements with enhanced photocurrent, supporting the reported electron-transfer pathway without demonstrating fuel production.^77^ Conversely, the formic-acid and water-to-H_2_/H_2_O_2_ systems quantify chemical products but involve different hydrogen sources and carbon architectures.^78,79^ The TiO_2_-based hybrid establishes improved photoelectrochemical response, although product-specific efficiencies cannot be inferred from photocurrent alone.^80^ Electrochemical CO_2_ reduction provides an additional example of productive Au–GQD catalysis, but does not establish a photochemical contribution.^81^ Accordingly, these studies support architecture-specific interfacial functions rather than a universal charge-transfer mechanism or a common performance ranking.

**Table 2:** Comparative experimental evidence linking Au–GQD architecture, optical and charge-transfer responses, and catalytic performance, with distinctions between solar-fuel demonstrations and supporting studies

Hybrid architecture	Application and operating basis	Reported performance or observation	Interfacial interpretation	Evidence scope and limitation	Ref.
Microplasma-prepared Au@GQD nanohybrids integrated with graphene	Broadband photodetection	Responsivity ≈10^3^ A W^−1^; detectivity ≈10^13^ Jones; rise/fall times: 65/53 ms	Au-associated absorption and hybrid integration support broadband photoresponse; strong fluorescence quenching observed	Supporting optoelectronic evidence; no chemical-product or fuel-generation measurement	74
AuNP/GQD dispersions with different AuNP concentrations	Plasmon-enhanced fluorescence and Cr^3+^ sensing	2.67-Fold fluorescence enhancement at low AuNP concentration; excitation feature at 421 nm at higher concentration	Concentration-dependent plasmonic coupling and proposed hot-electron contributions modify GQD emission	Supporting optical evidence; fluorescence enhancement does not establish productive redox-carrier utilization	75
AuNPs/G_6_/GQDs with varied GQD surface states	Fluorescence modulation and SERS	Surface-state-dependent fluorescence and SERS responses	GQD surface chemistry influences excited-state interactions and electromagnetic enhancement	Supporting interfacial evidence; neither hydrogen production nor water splitting evaluated	76
Au-nanocluster-sensitized GQDs	Photoelectric conversion	Photocurrent 2.69-fold and 4.80-fold that of Au nanoclusters and GQDs, respectively; quenched donor emission and shortened lifetime	Reported type-II alignment supports electron transfer from Au nanoclusters to GQDs	Steady-state and time-resolved evidence supports charge separation; no quantified chemical products	77
Au coordinated to thiol-functionalized S-doped GQDs	Visible-light-assisted formic-acid conversion to H_2_	TOF up to 112 h^−1^; catalyst recyclability reported	Thiol-containing sites anchor Au and provide a chemically defined catalytic interface	Direct H_2_-generation evidence; formic acid supplies hydrogen, so this is not overall water splitting	78
Graphene-wrapped Au derived from N-GQD polycondensation	Photocatalytic conversion of water to H_2_ and H_2_O_2_	H_2_: 65.6 µmol g^−1^ h^−1^; H_2_O_2_: 49.7 µmol g^−1^ h^−1^	*In situ* carbon-shell formation establishes Au–carbon contact; Au LSPR proposed to support photocatalysis	Direct product-generation evidence; final carbon phase is reconstructed graphene rather than preserved GQDs	79
GQD-grafted AuNPs on TiO_2_ with co-exposed {001}/{010} facets	Biased photoelectrochemical water-splitting configuration	0.535 mA cm^−2^ at 1.23 V *vs.* RHE, *versus* 0.267 mA cm^−2^ for pristine TiO_2_	Larger AuNPs favor plasmonic enhancement; GQD-mediated hot-electron extraction proposed	Approximately twofold photocurrent enhancement; current alone does not quantify H_2_ yield or Faradaic efficiency	80
N-GQD-wrapped single-crystalline AuNPs	Electrochemical CO_2_ reduction to CO	Maximum CO Faradaic efficiency: 93% at −0.25 V *vs.* RHE; CO formation begins at −0.15 V *vs.* RHE	Surface modification and Au–NGQD interactions enhance catalytic activity, supported by DFT interpretation	Direct electrochemical product evidence; no demonstration of photon-driven conversion	81

### 4.5. Cross-cutting evidence synthesis and maturity of the Au–GQD solar-conversion field

Taken collectively, the available evidence portrays Au–GQD research as a mechanistically promising but structurally fragmented field. The literature does not yet converge on a single material identity, because “Au–GQD hybrid” encompasses systems in which GQDs act as reductants, stabilizers, electron acceptors, surface ligands, optical modifiers, carbon precursors, or charge-extraction domains, while Au alternately functions as a catalytic centre, plasmonic absorber, sensitizer, structural template, or electron donor.^73,80^ This functional diversity broadens the design space, but it also weakens comparability because apparently similar hybrid labels can refer to fundamentally different interfaces and operating mechanisms.

A consistent trend is the transition from simple component combination toward deliberate control of interfacial function. Rapid and mild hybrid formation demonstrates that Au–GQD integration is synthetically accessible, whereas the optical studies show that neither Au content nor interfacial proximity produces a universally beneficial response.^75^ Depending on concentration, geometry, and surface-state distribution, coupling can favour electromagnetic enhancement, energetic-carrier transfer, fluorescence quenching, or dissipative relaxation.^76^ The type-II Au-nanocluster/GQD system further indicates that favourable energy-level alignment can translate interfacial coupling into enhanced charge separation,^77^ but this conclusion cannot be generalized without accounting for the surface-state heterogeneity identified elsewhere. The literature therefore converges on interfacial selectivity, rather than simple hybrid formation, as the central performance determinant.

A second field-level tension concerns structural persistence. Some systems preserve GQDs as electronically active interfacial components,^77^ whereas another uses them as precursors that evolve into larger graphene domains during catalyst construction.^79^ This distinction is scientifically important because performance attributed to quantum confinement or GQD surface states may instead originate from reconstructed carbon architectures. Structural verification before and after operation is therefore necessary to determine whether the active material remains an Au–GQD hybrid or becomes a different Au–carbon phase.

The evidence base also remains divided between direct solar-fuel studies and indirect optical or electronic demonstrations. Photodetection, fluorescence modulation, and spectroscopic energy transfer clarify possible interfacial pathways,^74^ but they do not establish chemical-energy conversion. Conversely, the hydrogen-production and water-splitting studies verify functional feasibility but use different reactions, architectures, illumination conditions, and activity metrics.^80^ These differences prevent meaningful performance ranking and obscure the relative contributions of plasmonic excitation, catalytic Au sites, GQD-mediated transport, and carbon reconstruction.

The current literature therefore supports a shift from composition-centred reporting toward function-resolved classification of Au–GQD systems. The most defensible framework would distinguish stable quantum-dot interfaces from transformed carbon architectures and separate optical enhancement from verified carrier utilization and chemical turnover.^25,67^ This synthesis identifies the maturity limits of the field and provides a natural basis for Section 5, where unresolved standardization, scalability, durability, and translational requirements can be addressed.

## 5. Unresolved challenges and future research priorities for Au–GQD solar-fuel systems

### 5.1. Resolving the identity of the active interface under operating conditions

The active Au–GQD interface should be identified under illumination, in the reaction medium, and, for photoelectrodes, at the operating potential. Measurements performed in a simplified environment provide useful mechanistic information but should not automatically be described as *operando*. A practical investigation should connect carrier generation, interfacial structural evolution, and product formation across complementary timescales. The following workflow is proposed for Au–GQD systems; methodological demonstrations in other material classes provide guidance rather than direct validation of Au–GQD mechanisms.

First, femtosecond-to-nanosecond transient absorption spectroscopy should compare excitation near the Au localized surface plasmon resonance with excitation preferentially absorbed by GQDs or the semiconductor support. Absorption spectra should guide wavelength selection, and excitation overlap must be quantified when selective pumping is incomplete. Broadband probing should resolve Au-associated transient features together with independently assigned acceptor-state signals. Evidence for injection should include the appearance of an acceptor population with kinetics consistent with donor-state depletion, supported by component controls and spectroelectrochemical assignments. Measurements over a low-fluence range are needed to identify nonlinear excitation and heating artifacts. Recent pump–probe measurements on gold-nanostar/poly(heptazine imide) composites demonstrate the feasibility of distinguishing excitation-dependent electron scavenging and hot-electron injection, although the resulting parameters cannot be transferred directly to Au–GQD interfaces.^82^

Second, nanosecond-to-second transient absorption or photoinduced absorption should track whether initially separated charges survive long enough to participate in chemistry. Time-resolved photoluminescence can complement these measurements, but emission quenching or shortened lifetime alone cannot distinguish productive transfer from nonradiative trapping. Carrier-population changes should therefore be compared with product-formation rates under matched illumination, electrolyte composition, temperature, and reactant concentration.

Third, dynamic structural measurements should use repeated light–dark cycles to separate reversible interfacial responses from irreversible reconstruction. *Operando* Au L_3_-edge X-ray absorption near-edge structure and extended X-ray absorption fine structure can probe changes in Au electronic state and local coordination, subject to sufficient Au loading and signal quality. Their acquisition times should be reported explicitly; conventional scans resolve accumulated structural changes rather than femtosecond injection. Concurrent or matched-condition Raman and attenuated-total-reflection infrared measurements can examine carbon-framework evolution, functional-group changes, and adsorbed intermediates. Beam-dose controls, probe-laser-power controls, and reference compounds are required because irradiation damage, local heating, and overlapping bands can mimic interfacial changes. These measurements are proposed tests of structural persistence, not established observations for every Au–GQD catalyst.

Fourth, photoelectrodes should be examined using chopped-light chronoamperometry, illuminated electrochemical impedance spectroscopy, and intensity-modulated photocurrent spectroscopy (IMPS). Measurements should span relevant potentials and illumination intensities after establishing steady operation. Under an appropriate kinetic model, IMPS can distinguish changes in effective surface-transfer and recombination rates; experimental studies on hematite demonstrate why improved photocurrent need not imply faster catalytic turnover.^83^ Impedance-derived parameters remain model-dependent, and a smaller fitted resistance alone does not establish transfer direction. Electrical measurements should be coupled with calibrated gas chromatography for H_2_/O_2_ and validated peroxide assays where relevant, allowing product-specific faradaic efficiencies and redox balances to be evaluated.

Finally, Au-only, GQD-only, support-only, physical-mixture, and chemically coupled controls should be compared under matched conditions. Temperature-matched dark experiments and wavelength-dependent product yields should accompany plasmonic assignments. A credible mechanism requires agreement between carrier dynamics, operating-state structure, electrochemical kinetics where applicable, and quantified products.

### 5.2. Benchmarking, standardization, and causal performance attribution

The absence of consistent evaluation criteria remains a major obstacle to comparing Au–GQD systems across photocatalytic and photoelectrochemical configurations. Activity values reported under different light sources, spectral distributions, reactor geometries, catalyst masses, electrode areas, sacrificial agents, and mass-transfer conditions cannot be interpreted on the same basis. A credible benchmarking framework must separate intrinsic interfacial activity from reactor-level effects and must distinguish light absorption from actual solar-to-chemical conversion.

Performance reporting should include photon-normalized metrics, wavelength-resolved response, product selectivity, faradaic efficiency where applicable, and full carbon or oxygen balance when side products may form. Apparent reaction rates should be accompanied by absorbed-photon information rather than incident-light intensity alone. For particulate systems, normalization exclusively by catalyst mass may overstate differences caused by dispersion or accessible surface area; for electrodes, geometric current density should be complemented by electrochemically active area and optical absorption under operating conditions. Stability should also be evaluated through retained conversion efficiency and preserved product selectivity, not only through structural appearance after cycling.^74,79^

Causal attribution requires appropriately designed controls. Au, GQDs, physical mixtures, chemically coupled hybrids, and interface-blocked analogues should be compared under identical conditions. Dark controls, thermal controls, wavelength-selective excitation, and scavenger-free operation are needed to differentiate photochemical conversion from heating, sacrificial chemistry, or uncontrolled side reactions. Where plasmonic effects are proposed, optical enhancement, hot-carrier transfer, and temperature rise should be independently quantified.

Quantitative separation of plasmonic near-field enhancement from hot-electron injection remains unresolved in the Au–GQD systems considered here. Fluorescence enhancement, donor-emission quenching, and increased photocurrent probe different stages of excitation and charge utilization; none independently provides the percentage contribution of either pathway to fuel production.^74–77,80^ A prospective discrimination strategy should compare direct-contact hybrids with electronically blocking, optically characterized spacer controls and Au-free references under matched excitation, temperature, and reaction conditions. Spacer thickness must be varied systematically because suppressing electron tunneling also changes the near-field strength, dielectric environment, and accessibility of catalytic sites.

Product-based apparent quantum yields should be measured alongside optical absorption and transient carrier populations. For H_2_ formation, the incident-photon-based apparent quantum yield is AQYH_2_ = 2N(H_2_)/Nincident photons × 100%, where both quantities refer to the same irradiation interval. Electromagnetic simulations can estimate changes in semiconductor absorption, while transient spectroscopy can constrain the appearance and survival of injected carriers. These measurements should be interpreted jointly: enhanced absorption does not establish productive charge utilization, and injection yield does not equal fuel-generation yield. Because the pathways can interact through trapping, recombination, and surface kinetics, subtracting the activity of a spacer-containing control from that of a direct-contact hybrid does not necessarily isolate a unique hot-electron contribution. Until matched product-resolved datasets and validated kinetic models become available, numerical partitioning should remain explicitly undetermined.

Plasmonic excitation and photothermal acceleration are not mutually exclusive: relaxation of plasmon-generated carriers ultimately contributes to heat, while energy or charge transfer may compete with that relaxation. Consequently, a reaction action spectrum resembling Au absorption, or an increased rate under resonant illumination, does not independently establish a nonthermal mechanism. Evaluation should combine wavelength-resolved product yields, carrier-sensitive spectroscopy, and temperature-matched dark kinetics. Temperature measurements must represent the catalyst environment as closely as possible, since bulk-electrolyte readings may miss local gradients. Light-intensity dependence is informative but cannot independently distinguish heating from carrier-mediated chemistry. Where local temperature and productive carrier utilization remain unresolved, the measured enhancement should be described as light-assisted, with photothermal and photochemical contributions explicitly left unpartitioned.

Standardization must also extend to material descriptors. Reporting average particle size and elemental composition is insufficient without distributions of Au size, GQD dimensions, surface functionality, contact configuration, and interfacial bond identity. A minimum reporting protocol linking synthesis variables to physicochemical descriptors and performance metrics would substantially improve reproducibility. Once comparable and causally interpretable datasets become available, the field can progress from isolated performance claims toward predictive structure–function relationships. Such relationships, however, will remain incomplete unless long-term stability and scalable production are addressed simultaneously.

### 5.3. Stability, scalable manufacturing, and sustainability constraints

The translation of Au–GQD hybrids from laboratory studies to sustained solar-fuel operation depends on maintaining interfacial function under continuous photon exposure, electrochemical bias, reactive intermediates, and fluctuating environmental conditions. Stability cannot be defined solely as the retention of bulk morphology. Small changes in GQD surface oxidation, Au coordination, interfacial bonding, or ligand coverage may alter the direction and rate of charge transfer before visible structural degradation occurs. Functional stability should therefore be assessed through time-resolved changes in work function, charge-transfer resistance, optical response, product selectivity, and interfacial chemical state.

Light-induced reconstruction should be distinguished from transformations deliberately introduced during catalyst synthesis. In ref. 79, N-GQDs undergo polycondensation into graphene during preparation; this establishes a change in material identity but does not demonstrate progressive GQD degradation during photoelectrochemical cycling. Nevertheless, oxidation of the carbon framework, loss or rearrangement of anchoring groups, and detachment of GQDs are plausible degradation pathways that require direct evaluation under the operating electrolyte. Such changes could alter conjugation, interfacial dipoles, carrier trapping, and Au attachment, progressively reducing selective charge extraction. Related metal-nanocluster studies further demonstrate that the operating Au species may differ from the initial photosensitizer, emphasizing the importance of identifying reconstructed states rather than interpreting activity solely from the starting structure.^86^

Au aggregation represents a distinct potential failure pathway. Particle growth or coalescence can decrease accessible metal surface area, modify plasmon resonance and local fields, and disrupt the distribution of Au–GQD contacts. Electrolyte pH, ionic strength, specifically adsorbing ions, and applied potential may affect ligand binding and colloidal or electrode stability. Their effects should therefore be assessed experimentally rather than assumed to be transferable between electrolytes.

Long-term testing should combine continuous operation with repeated light–dark and potential cycles in a fully specified electrolyte. Product rates, selectivity, and faradaic efficiency should be followed alongside photocurrent, because stable current can conceal changing parasitic reactions. Time-dependent optical spectra, Raman measurements, Au coordination analysis, and before/after particle-size distributions can test whether performance loss accompanies carbon reconstruction or Au growth. Analysis of dissolved Au and released carbon species can further distinguish leaching and detachment from aggregation. Protective coatings and stronger anchoring should be evaluated together with transport and catalytic accessibility, since stabilization may also introduce charge-transfer or mass-transfer barriers.

Several degradation pathways require systematic separation. GQD oxidation can modify edge states and reduce electronic connectivity, whereas excessive reduction may remove functional groups required for Au anchoring. Au nanoparticles may aggregate, detach, become chemically passivated, or develop adsorbate-covered surfaces that alter catalytic activity.^76,78^ Organic linkers and surface ligands can stabilize colloids but may degrade under illumination or block reactive sites. In multicomponent photoelectrodes, mechanical delamination and electrolyte-induced swelling introduce additional failure modes. Accelerated tests should therefore be designed around realistic photon flux, electrolyte composition, temperature variation, and start–stop operation rather than relying exclusively on short continuous runs.

Scalable synthesis imposes a separate set of constraints. Batch methods that provide acceptable hybrids at small scale may produce broad particle-size distributions, variable GQD chemistry, and nonuniform Au loading when precursor concentration and mixing conditions are increased. Continuous-flow processing, controlled nucleation, and inline optical or electrochemical quality monitoring may offer better reproducibility, but these routes must preserve interfacial specificity rather than merely increase throughput.

Sustainability also requires attention to Au utilization, recovery, and life-cycle impact. Because Au is costly and resource-intensive, future designs should maximize atom efficiency, catalytic accessibility, and recyclability rather than increasing metal loading. Synthetic routes should minimize toxic reductants, excess ligands, and purification demands. Accordingly, scale-up should be treated as a materials-function problem rather than a manufacturing afterthought. The ability to retain defined interfaces at larger scale will determine which design concepts are sufficiently robust for integration into next-generation solar-fuel devices.

### 5.4. Next-generation architectures and system-level integration

Future Au–GQD systems should move beyond binary hybrid formation toward architectures in which each component is assigned a defined optical, electronic, and catalytic function. The primary design objective should be directional control over the full conversion pathway: photon capture, excited-state generation, carrier extraction, transport, surface accumulation, and redox turnover. This requires spatially resolved architectures rather than uniform mixing. Au domains responsible for plasmonic excitation may need to be separated from catalytic Au sites, while GQD populations optimized for light absorption may differ from those optimized for charge transport or chemical anchoring.

One promising direction is the development of graded or asymmetric interfaces that impose a preferred carrier-transport direction. Such systems could combine controlled work-function variation, selective surface functionalization, and spatial separation of reduction and oxidation sites. The challenge is to create energetic asymmetry without introducing transport barriers or excessive interfacial resistance. Three-dimensional porous photoelectrodes may improve photon utilization and molecular transport, but their geometry must avoid optical shielding, inaccessible pores, and nonuniform electrical connectivity.^75,80^

The design of GQDs should also become more state-specific. Rather than using broad distributions of size and functionality, future synthesis should target narrow populations with controlled edge chemistry, dopant configuration, and surface-state density. Similarly, Au should be selected according to its intended role, distinguishing atomically dispersed sites, nanoclusters, and plasmonic nanoparticles rather than treating all Au domains as functionally equivalent. This functional classification may enable hybrid systems in which light harvesting and catalytic turnover are optimized independently.

Data-driven approaches could support this transition if they are grounded in physically meaningful descriptors. Models should incorporate interfacial bond identity, work-function difference, optical overlap, carrier lifetime, surface-state distribution, adsorption energetics, and stability indicators rather than relying only on synthesis conditions and final activity. Closed-loop experimentation could then identify compositions and architectures that balance multiple objectives, including efficiency, selectivity, durability, and Au utilization. The field will advance most effectively when material design is coupled to reactor and device engineering from the outset. This systems-level perspective provides the final bridge from mechanistic understanding to practically relevant solar-to-chemical conversion.

Related artificial photosystems offer useful design principles for Au–GQD hybrids. Spatial confinement and nanocluster sensitization highlight the importance of organized interfaces and productive charge separation.^84,85^ Structural reconstruction studies further demonstrate that operating metal species can differ from their initial configurations.^86^ Photoelectrochemical CO_2_ conversion provides a framework for integrating light harvesting, carrier transport, and fuel production.^87^ Directional electron-transfer chains and polymer-mediated tunneling illustrate complementary approaches to controlling interfacial charge migration.^88,89^ Alloy nanocluster research identifies composition as an additional variable for tuning photocatalytic behavior and hydrogen generation.^90,91^ Self-assembly offers routes for arranging these functional components.^92^ These related systems motivate Au–GQD interface engineering, but their applicability requires independent validation under relevant illumination, electrolyte, bias, and reaction conditions.

Additional related systems broaden the design context for Au–GQD interfaces. AgCu-modified BiFeO_3_ combines plasmonic and piezophototronic effects for solar nitrogen reduction, illustrating the need to distinguish contributions from multiple excitation mechanisms.^94^ Au@UiO-66-NH_2_ provides a complementary example of plasmonic Au integration for visible-light-driven H_2_O_2_ and H_2_ production, with catalyst preparation influencing performance.^95^ The rGO-containing NH_2_-MIL-125(Ti)/ZIF-67 architecture offers a reported Z-scheme framework for discussing carbon-mediated charge dynamics,^96^ while mesoporous rGO-integrated Ce/Ti nanorods connect carbon incorporation and structural engineering with visible-light hydrogen generation.^97^ These studies inform interfacial design and comparative mechanistic analysis; their extended carbon structures, host materials, or additional energy inputs preclude direct transfer of their mechanisms and efficiencies to Au–GQD systems.

Broader advances in interfacial photocatalysis provide additional context for Au–GQD design. NiFe layered double hydroxide/g-C_3_N_4_ heterostructures highlight strategies involving heterointerface construction, defect engineering, and charge-transfer pathways for solar-driven hydrogen generation.^98^ Research on layered double hydroxide–carbonaceous assemblies further connects carbon integration with water-splitting and energy-storage applications, providing a comparative framework for evaluating the function of carbon components.^99^ Complementarily, CdSe quantum dots coupled to CuS nanosheets enhance photocatalytic H_2_O_2_ production through oxygen reduction, illustrating a distinct solar-to-chemical pathway rather than overall water splitting.^100^ These related systems motivate careful coordination of light absorption, carrier separation, and surface reactions, while their different compositions and reaction conditions preclude direct attribution of their mechanisms or performance to Au–GQD interfaces.

## 6. Conclusion

Au–GQD hybrid systems occupy a distinctive position in solar-driven energy conversion because they combine tunable carbon quantum states with the electronic, catalytic, and plasmonic characteristics of nanoscale gold. The central contribution of this review is to frame their performance not as a simple consequence of combining two functional materials, but as the outcome of interdependent optical, electronic, interfacial, and chemical processes. The decisive variable is the nature of the operating interface: its electronic-state distribution, bonding configuration, structural persistence, and ability to direct carriers toward productive redox pathways. The field's principal limitation is not the absence of plausible mechanisms, but the difficulty of separating them and linking them causally to chemical conversion.

Optical enhancement, hot-carrier injection, charge trapping, catalytic turnover, and structural reconstruction are frequently entangled, while inconsistent reporting prevents rigorous comparison across systems. Progress therefore requires *operando* identification of active states, standardized photon- and product-based metrics, controlled interface libraries, and stability assessment under realistic operating conditions. A credible development pathway should combine narrow material distributions, function-specific Au and GQD domains, directional interface design, low-Au architectures, and scalable synthesis with real-time quality control. Under this framework, future advances will be judged not by isolated activity gains, but by reproducible control over the complete solar-to-chemical conversion sequence.

## Conflicts of interest

There are no conflicts to declare.

## Data Availability

No primary research results, software or code have been included and no new data were generated or analysed as part of this review.
